# Emerging roles of exosomes in the diagnosis and treatment of kidney diseases

**DOI:** 10.3389/fphar.2025.1525314

**Published:** 2025-04-16

**Authors:** Huanhuan Cao, Zixi Li, Jiajia Ye, Yi Lv, Chun Zhang, Tao Liang, Yumei Wang

**Affiliations:** ^1^ Department of Nephrology, Union Hospital, Tongji Medical College, Huazhong University of Science and Technology, Wuhan, China; ^2^ Department of Clinical Laboratory, Traditional Chinese and Western Medicine Hospital of Wuhan, Tongji Medical College, Huazhong University of Science and Technology, Wuhan, China; ^3^ Department of Clinical Laboratory, Union Hospital, Tongji Medical College, Huazhong University of Science and Technology, Wuhan, China

**Keywords:** exosomes, kidney disease, diagnosis, biomarkers, treatment

## Abstract

The complex etiology and spectrum of kidney diseases necessitate vigilant attention; the focus on early diagnosis and intervention in kidney diseases remains a critical issue in medical research. Recently, with the expanding studies on extracellular vesicles, exosomes have garnered increasing interest as a promising tool for the diagnosis and treatment of kidney diseases. Exosomes are nano-sized extracellular vesicles that transport a diverse array of bioactive substances, which can influence various pathological processes associated with kidney diseases and exhibit detrimental or beneficial effects. Within the kidney, exosomes derived from the glomeruli and renal tubules possess the ability to enter systemic circulation or urine. The biomarkers they carry can reflect alterations in the pathological state of the kidneys, thereby offering novel avenues for early diagnosis. Furthermore, research studies have confirmed that exosomes originating from multiple cell types exhibit therapeutic potential in treating kidney disease; notably, those derived from mesenchymal stem cells (MSCs) have shown significant treatment efficacy. This comprehensive review summarizes the contributions of exosomes from different cell types within the kidneys while exploring their physiological and pathological roles therein. Additionally, we emphasize recent advancements in exosome applications for the diagnosis and treatment of various forms of kidney diseases over the past decades. We not only introduce the urinary and blood biomarkers linked to kidney diseases found within exosomes but also explore their therapeutic effects. Finally, we discuss existing challenges and future directions concerning the clinical applications of exosomes for diagnostic and therapeutic purposes.

## 1 Introduction

Kidney diseases are varied and prevalent across the globe, impacting the quality of life and health of millions of individuals. These conditions present a significant challenge to healthcare systems worldwide. Severe kidney disease can progress to end-stage renal disease (ESRD); therefore, early recognition and proactive interventions are essential for mitigating the global burden of kidney disease (Eknoyan et al., 2004). Urinalysis and blood tests have been acknowledged as critical indicators for assessing and monitoring renal function, including proteinuria and serum creatinine levels. However, their utility in evaluating renal disease is limited by issues such as specificity and sensitivity as they can be influenced by various physiological and pathological factors, often lagging behind the onset of early kidney damage (Aklilu, 2023). Renal puncture biopsy remains the gold standard for diagnosing most renal diseases; however, it is an invasive procedure that carries risks such as bleeding and infection during puncture, making it unsuitable for routine screening. Consequently, identifying non-invasive and sensitive biomarkers for the diagnosis of renal diseases is imperative (Chen et al., 2018).

Exosomes play a pivotal role in renal physiology and pathology, acting as essential mediators of intercellular communication. Exosomes are present in nearly all cell types and body fluids, including plasma, urine, saliva, breast milk, cerebrospinal fluid, and ascites. Notably, certain components of exosomes, such as microRNAs (miRNAs), have the potential to serve as biomarkers for early-stage renal pathology. The progression of kidney disease can be monitored by assessing alterations in the quantity or composition of exosomes (Grange and Bussolati, 2022). Furthermore, due to their natural properties as vesicular carriers, exosomes can specifically target damaged kidney cells by delivering proteins, RNAs, and therapeutic agents to facilitate cellular repair and regeneration. The unique characteristics of exosomes present promising opportunities for the development of innovative therapeutic strategies aimed at treating kidney diseases.

This review discusses the relevant characteristics of exosomes, summarizes their mechanisms in various renal diseases based on existing studies, and investigates their potential as biomarkers and drug delivery systems for the diagnosis and treatment of renal disorders.

## 2 Biological characteristics of exosomes

### 2.1 Biogenesis, secretion, and structure of exosomes

Johnstone et al. (1987) were the pioneers in observing that amniotic reticulocytes can release membrane vesicles during their study of reticulocyte maturation, marking one of the early discoveries related to exocytosis. Initially, extracellular vesicles (EVs) garnered limited interest and were primarily regarded as a mechanism for cells to eliminate waste products. However, with ongoing research, the biological characteristics and significance of exosomes have gradually gained recognition and appreciation. Exosomes are a specific class of extracellular vesicles, ranging from 30 to 200 nm in diameter, produced by various cell types, and widely distributed across biological fluids such as blood, urine, saliva, pleural fluid, cerebrospinal fluid, breast milk, and semen. Exosome biogenesis involves four critical steps, namely, cargo sorting into multivesicular bodies (MVBs), MVB formation and maturation, MVB transport, and the fusion of MVBs with the plasma membrane (Han et al., 2022). Cargo sorting is responsible for targeting specific molecules and proteins within MVBs. The synthesis of exosomes typically begins at the early endosome, which originates from the inward budding of the cell membrane. At this stage, the endocytosed cargo undergoes primary sorting and fate determination. This process generally leads to three potential outcomes: recycled cargoes are localized to the peritubular region of the endosome before being separated and fused with either the Golgi apparatus or the plasma membrane of recycling endosomes. In contrast, the unrecovered cargoes become concentrated in the central region of the early endosome as it follows a maturation pathway toward late endosomes. Within late endosomes, some components of the endocytosed material are further sorted and processed, which ultimately results in MVB formation (Krylova and Feng, 2023). Typically, MVBs face one of the two fates: they may either bind to lysosomes for degradation due to their ubiquitinated contents or fuse with cytoplasmic membranes to release their contents into extracellular space (Doyle and Wang, 2019a).

**FIGURE 1:**
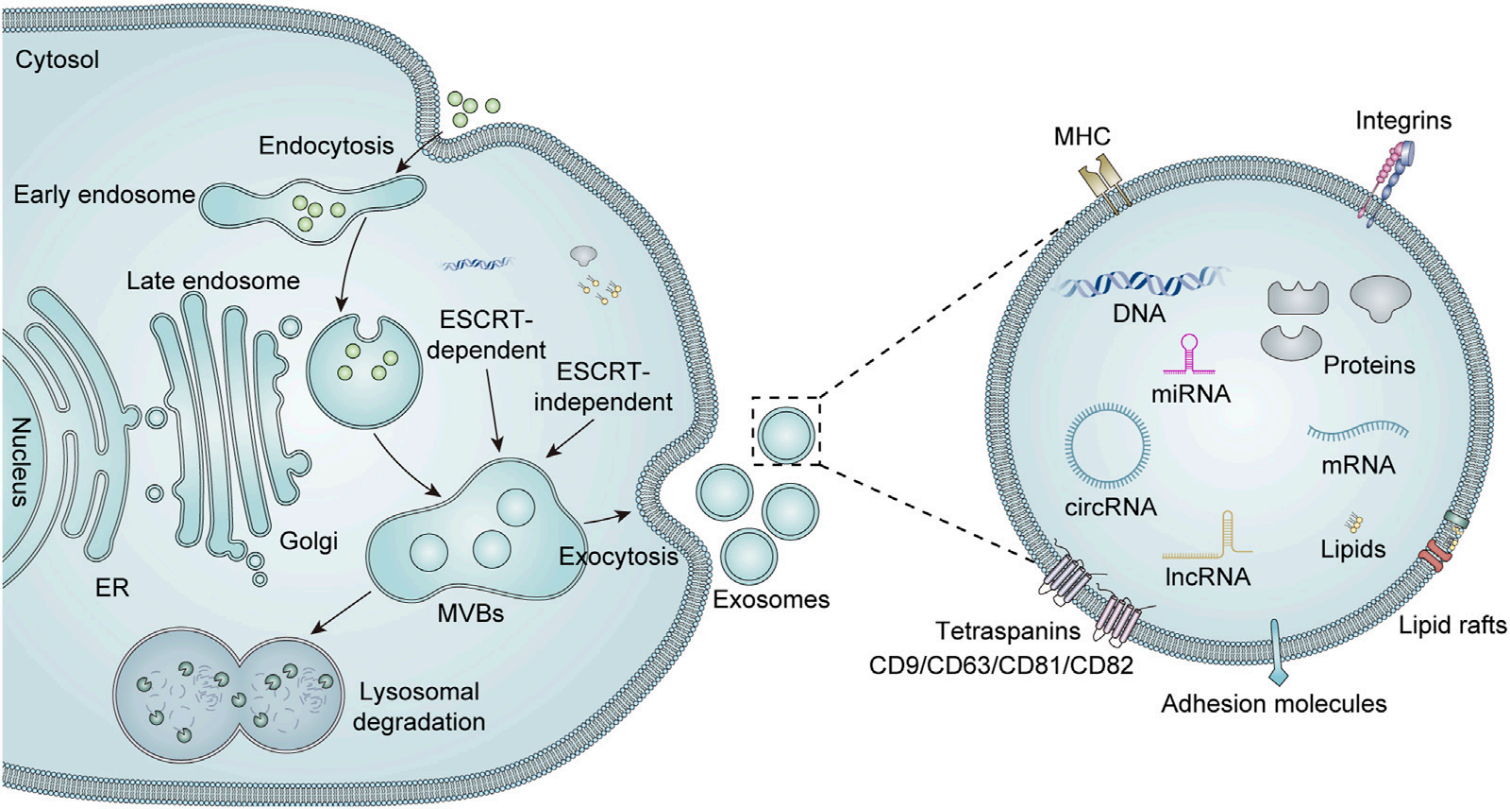
Biogenesis, secretion, and structure of exosomes. Extracellular components can enter the cell via intracellular endocytosis. The biogenesis of exosomes begins with the formation of early endosomes, which are formed by invagination of the cytoplasmic membrane. These early endosomes subsequently mature into late endosomes, which can evolve into MVBs through the accumulation of numerous intraluminal vesicles (ILVs). There are two recognized pathways for regulating MVBs, namely, endosomal sorting complexes required for transport (ESCRT)-dependent and ESCRT-independent mechanisms. Mature MVBs may either fuse with lysosomes for degradation or are transported to the cell membrane to merge with the plasma membrane, thereby releasing their luminal vesicles as exosomes into the extracellular space. Exosomes are mainly enriched with a variety of biologically active substances, including lipids, proteins (e.g., major histocompatibility complex (MHC), integrins, tetra-transmembrane proteins, and heat shock proteins), and nucleic acids (DNA, mRNA, miRNA, lncRNA, and circRNA).

The structural composition of exosomes is illustrated in Figure 1. Exosomes possess a well-defined membrane structure primarily composed of proteins and phospholipids. They are characterized by various surface proteins, including tetraspanins (CD9, CD63, CD81, and CD82), adhesion proteins, integrins, and glycoproteins. Certain specific surface proteins can be utilized to identify exosomes after isolation, thereby confirming the presence of exosome-like vesicles. Furthermore, the surface of exosomes contains distinct marker molecules associated with their parental cells. These markers can facilitate the identification or selection of particular exosome populations and enable the screening for disease-related biomarkers within these populations or the analysis of their functional roles. The exosomal components are diverse and capable of carrying an array of cargo molecules such as nucleic acids (including DNA, miRNAs, mRNA, siRNA, circular RNA, rRNA, and long non-coding RNA), proteins (e.g., Alix proteins, TSG101, heat shock proteins, and cytoskeletal proteins), and lipids (Liang et al., 2023). The cargoes carried by exosomes are closely linked to those of their parent cells. Consequently, there exists variability in the cargoes derived from different cell sources, which contributes to heterogeneity. Additionally, both the composition and quantity of exosomal cargoes may alter under pathological conditions. Qualitative and quantitative analyses of the contents within exosomes can be performed using techniques such as nucleic acid sequencing, proteomics, and lipidomics (Liu et al., 2024).

### 2.2 Characteristics of exosomes

As nanoscale EVs, exosomes have unique and superior properties that render them highly valuable for biomarker screening and clinical diagnosis. First, exosomes feature a membranous structure that ensures stability across various body fluids, thereby protecting their cargoes from enzymatic degradation in the external environment. Second, the contents of exosomes—comprising nucleic acids, proteins, and lipids—exhibit alterations associated with disease states. Moreover, exosomes can be isolated from diverse types of body fluid samples through non-invasive methods, facilitating dynamic monitoring of diseases (Liang et al., 2023). Given the ease of sample acquisition, numerous studies have sought to identify suitable exosomal biomarkers from blood and urine samples for the early diagnosis and therapeutic monitoring of kidney diseases.

Numerous studies have demonstrated that urinary exosomes have great potential for the early diagnosis of a wide range of renal and urological diseases (Harita, 2024). Urinary exosomes possess multiple properties and originate from various cellular sources. They can be produced by different cell types within the genitourinary tract, including glomerular cells, tubular cells, thylakoid cells, and podocytes. These exosomes reflect the physiological and pathological states of the genitourinary system (Grange et al., 2023). The diverse origins of these cell-derived exosomes enable them to carry nucleic acids and proteins from their parent cells, thereby providing insights into the physiological and pathological changes occurring in kidney tissue. Consequently, urine-derived exosomes have emerged as powerful non-invasive diagnostic biomarkers for kidney disease.

Based on these characteristics, exosomes are increasingly recognized as effective drug carriers for targeted drug delivery. Exosomes are small in size, abundant in quantity, low in immunogenicity, and possess significant stability. Additionally, they facilitate the transport of materials and the transfer of information between cells. The drugs carried by exosomes can be directed to specific target cells through the artificial loading of small-molecule drugs or modifications to the exosomal membrane. This targeted approach to drug delivery is expected to enhance medication efficacy for patients, decrease administration frequency, and minimize systemic side effects (Sharma et al., 2024). In summary, exosomes represent a promising next generation of revolutionary drug carriers with considerable potential and numerous advantages as therapeutic agents and delivery systems.

## 3 Regulation of exosome secretion and its interaction with target cells

As membranous vesicles that play an important role in intercellular communication, the secretion of exosomes is regulated by a variety of factors, including molecular intervention (such as lipopolysaccharides, interferons, cytokines, and growth factors), culture conditions, physicochemical factors, and nanoparticles (Gurunathan et al., 2021b). For example, it has been suggested that the addition of certain small molecule regulators to the medium, such as the treatment of mesenchymal stem cells (MSCs) with a combination of N-methyldopamine and norepinephrine, can promote the production and secretion of exosomes (Wang et al., 2020). Similarly, there is increasing evidence that changes in the culture microenvironment, such as hypoxic preconditioning (Jiang et al., 2022), acidic environment (Logozzi et al., 2018), laser irradiation (Bagheri et al., 2018), mechanical stimulation (Guo et al., 2021), and serum deprivation (Haraszti et al., 2019) , can regulate the secretion of exosomes and change their contents, thereby affecting their biological function. In recent years, it has been reported that various nanoparticles, such as platinum nanoparticles, can enhance the biogenesis and secretion of exosomes (Gurunathan et al., 2021a). The physicochemical properties of micro/nano-textured hierarchical titanium topographies promote the proliferation of bone marrow mesenchymal stem cells (BMSCs), thus promoting the synthesis and secretion of BMSC-derived exosomes (Zhang Z. et al., 2021). In addition, some other biological materials, such as bioglass, can also promote the secretion of exosomes (Wu et al., 2020). In conclusion, exosomes are promising therapeutic tools for the treatment of various diseases, and an in-depth study of the related factors affecting the secretion of exosomes will help better understand their potential applications in disease diagnosis, therapy, and biomarker development.

Exosomes can be secreted by all types of living cells and are widely distributed in body fluids. They can transfer a variety of bioactive molecules, including nucleic acids, proteins, and lipids, to target cells, affect various biological processes of target cells, and ultimately affect the biological activity of target cells. Currently, it is believed that there are three types of interaction mechanisms between exosomes and target cells. (1) Direct membrane fusion: exosomes regulate target cells by directly fusing with the cell membrane and releasing their contents directly into the cell (Mori et al., 2019). (2) Endocytosis: target cells take up exosomes through endocytosis, and their contents are released or reprocessed within the cell, thereby regulating cell function. The endocytosis includes clathrin-independent and clathrin-dependent endocytosis processes. In addition, the clathrin-independent pathways are divided into lipid raft-mediated and caveolin-mediated endocytosis, phagocytosis, and micropinocytosis (Wu et al., 2021). (3) Receptor–ligand interaction: proteins, lipids, or lipoproteins on the membrane of exosomes can bind to specific receptors on the target cell membrane, triggering cell signaling pathways. It includes four transmembrane proteins, integrins, major tissue compatibility complex (MHC), intercellular adhesion molecule-1 (ICAM-1), proteoglycans, and lectins (Mulcahy et al., 2014).

## 4 Exosome isolation and purification techniques

In order to fully utilize the potential of exosomes in biomedical research, efficient isolation and purification techniques are essential (He et al., 2018). In recent years, with the deepening of exosome research, several methods have been proposed. However, various methods have different advantages and limitations. Several common techniques for exosome isolation and purification are described in Table 1.

**TABLE 1:** Advantages and limitations of different exosome isolation and purification methods.

Method	Advantage	Limitation	Reference
Ultracentrifugation	Low cost, easy to operate, reproducible, and stable	Time-consuming, low yield, and may damage the structure of exosomes	Lai et al. (2022)
Polymer precipitation	Simple and fast operation without expensive equipment	Low yield, poor purity, and impact on downstream analyses	Doyle and Wang (2019b)
Size exclusion chromatography	High efficiency, high purity, and the preservation of exosome integrity and bioactivity	Time-consuming, low total yield, and suitable for small samples	Liu et al. (2022a)
Ultrafiltration	Simple, cost-effective, and efficient	Membrane pore clogging and exosome loss	Yang et al. (2020)
Immunoaffinity	High specificity, high purity, and effective in reducing protein interference	High cost of antibodies and small sample size to process	Alzhrani et al. (2021)
Microfluidic technology	High efficiency, purity, and integration	Technically complex	Li et al. (2021b)

### 4.1 Ultracentrifugation

Ultracentrifugation is the most commonly used and classic method, which is regarded as the ‘gold standard’ for exosome extraction. Its basic principle is to take advantage of the difference in settling speeds of different particles under the action of centrifugal force. Ultracentrifugation is widely used for the isolation, purification, and functional study of exosomes, which offers advantages such as low cost, easy operation, good reproducibility, and stability. However, it is time-consuming, has a low yield, and may damage the structure of exosomes (Lai et al., 2022).

### 4.2 Polymer precipitation

The basic principle of polymer precipitation is to use polymers to interact with the water molecules surrounding the exosomes to form a hydrophobic microenvironment, thereby precipitating the exosomes. Polyethylene glycol (PEG) is the polymer most commonly used to precipitate exosomes. This method of isolation is simple and fast and does not rely on expensive equipment. However, this method is also capable of precipitating non-exosomal proteins, resulting in low yields and poor purity of the isolated exosomes (Doyle and Wang, 2019b).

### 4.3 Size exclusion chromatography

Size exclusion chromatography (SEC) is based on the size difference in molecules in gel packing. During separation and extraction, large molecules cannot enter the gel pores and are quickly eluted along the gap between the porous gel and the mobile phase, whereas small molecules are retained in the gel pores longer and eluted for a longer period of time. Exosome molecules are larger than normal protein molecules and, therefore, will be eluted first, resulting in the isolation of high-purity exosomes. Compared to centrifugation, SEC has higher separation efficiency and purity, and it can maintain the integrity and biological activity of the isolated exosomes. However, due to its time consuming nature and lower total yield, SEC is only suitable for exosome separation of small samples (Liu W.-Z. et al., 2022).

### 4.4 Ultrafiltration

Ultrafiltration is a technique in which exosomes are selectively separated by ultrafiltration membranes based on molecular particle size. Based on this principle, many simple and convenient ultrafiltration devices have been developed, such as microfilters configured in series and continuous ultrafiltration. Ultrafiltration is simple, economical, and efficient, but it also has its drawbacks. One of the prominent problems is the clogging of membrane pores, leading to a significant reduction in the service life of ultrafiltration membranes. More efficient forms of ultrafiltration are constantly being optimized to address these problems. For example, tangential flow filtration has become one of the main methods for rapid exosome isolation as it can continuously wash and flush the membrane surface, keeping it clean and functioning efficiently compared to traditional dead-end filtration (Yang et al., 2020).

### 4.5 Immunoaffinity

Immunoaffinity is a highly specific technique for exosome isolation and purification. It utilizes the affinity between antibodies and specific molecules on the exosome surface (e.g., tetraspanin proteins) to achieve selective capture and isolation of exosomes. Methods of exosome enrichment based on this principle are constantly being developed, such as enzyme-linked immunosorbent assays (ELISAs), immunomagnetic bead assays, and affinity peptides (Li et al., 2017). The immunoaffinity method is superior to ultracentrifugation in terms of sample volume required, purity, and yield. For example, it has been demonstrated that the immunoaffinity method can produce similar results with less sample volume, better morphological integrity of exosomes, and higher specificity than ultracentrifugation. The immunoaffinity method offers advantages such as high specificity, high purity, and the ability to effectively reduce protein interference, but it also has limitations such as high cost of antibodies, small sample size it can process, and its unsuitability for high-throughput separation (Alzhrani et al., 2021).

### 4.6 Microfluidic technology

Recently, with the continuous development of the field, the traditional exosome separation methods have struggled to meet the current growing demands of scientific research. Emerging exosome separation technology continues to emerge and gradually become the current hot spot of exosome separation research, one of which is based on the microfluidic exosome separation technology. It provides a new solution for the efficient and automated capture and purification of exosomes. The main feature of microfluidics is the precise manipulation of fluids in the micro- and nanoscale space and the use of exosome size, surface charge, and other physicochemical properties for the efficient separation and enrichment of exosomes (Al-Madhagi, 2024). Microfluidics-based assays offer advantages such as high efficiency, purity, and integration. They offer great potential for the discovery of exosomal biomarkers, diagnosis, and prognosis of diseases by integrating purification and enrichment of exosomes into microfluidic platforms (Li S. et al., 2021).

Exosomes have a large amount of genetic information and biomolecules derived from parental cells and play an important role in human physiological or pathological processes. The enrichment of high-purity exosomes from body fluids is a prerequisite for biofunctional and clinical application studies. It has been reported in the literature that combining different isolation methods can improve exosome isolation efficacy. Ultracentrifugation coupled with SEC (UC-SEC) has been identified as the best method for producing high-purity exosomes from the same volume of urine (50 mL) (Gheinani et al., 2018). Meanwhile, more efficient and optimized separation techniques have been successfully developed based on various traditional separation methods. For example, it has been reported that compared to the traditional ultracentrifugation method, the optimized ultrafiltration (OUF) method offers higher purity, yield, and biostability. Moreover, the higher miRNA expression in exosomes isolated by OUF may be suitable for large-scale extraction of clinical urine samples and subsequent experiments (He et al., 2019). In conclusion, with the rapid progress of exosomes in the diagnosis and treatment of clinical diseases, there is an urgent need to develop reproducible and scalable methods for the batch preparation of high-quality exosomes to meet clinical needs.

## 5 Exosomes from different sources in the kidney

**FIGURE 2:**
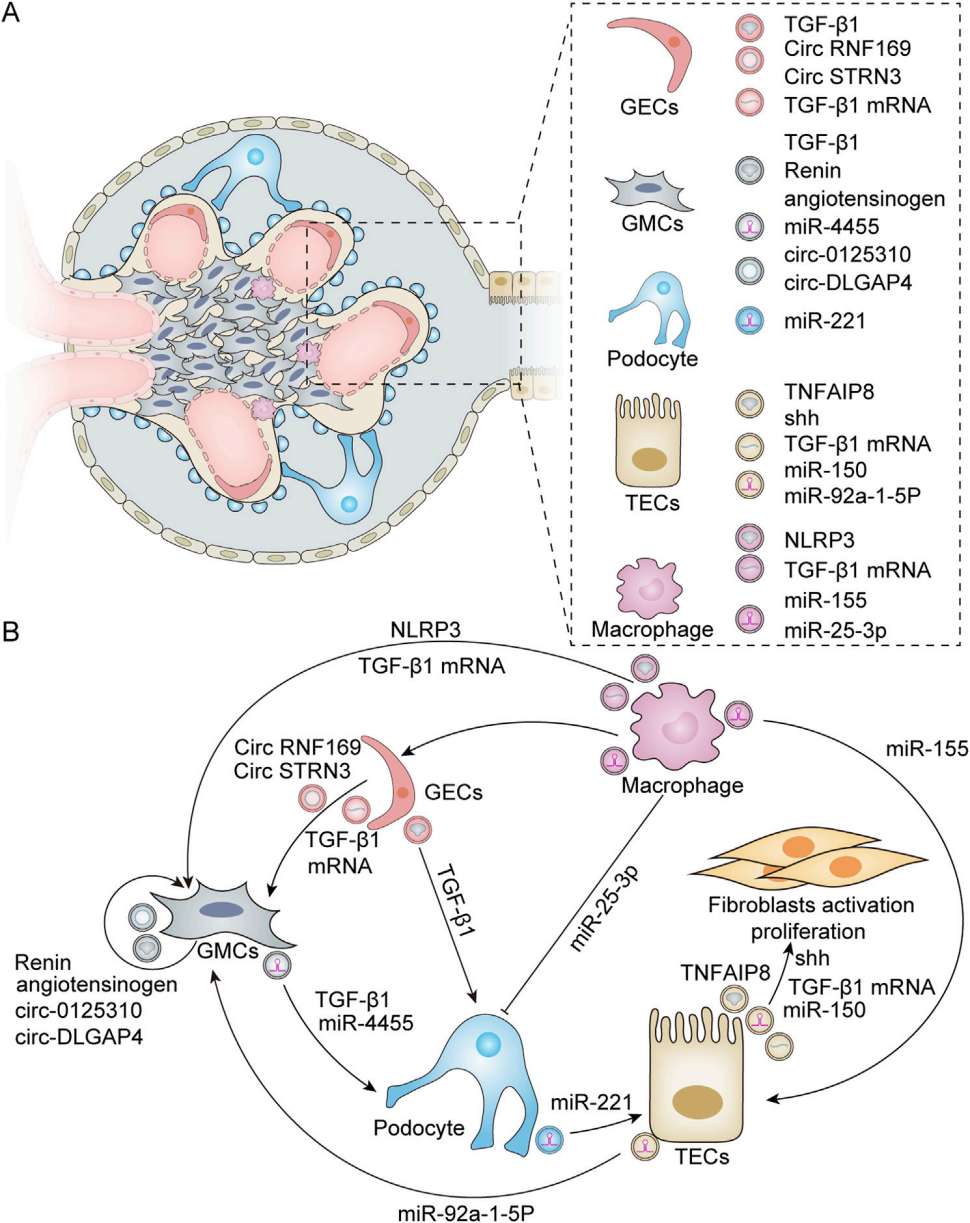
Exosomes derived from various renal resident cells and their crosstalk. **(A)** Exosomes derived from different renal resident cell types. Exosomes can derive from a variety of cell types within the kidney, including GECs, GMCs, podocytes, TECs, and macrophages. These exosome-derived biomolecules play a crucial role in modulating renal cell–cell interactions. **(B)** Exosome-mediated intercellular crosstalk among various renal resident cell origins. For GECs, HG treatment leads to the activation of GMCs by secreting exosomes enriched with TGF-β1 mRNA, circRNF169, and circSTRN3 (Wu et al., 2016; Ling et al., 2019). In addition, HG can induce GECs to release exosomes containing TGF-β1 to stimulate podocytes for EMT and dysfunction (Wu et al., 2017). Additionally, GMCs possess self-activation capabilities due to the active substances they produce. Exosomes released from HG-stimulated GMCs exhibit elevated levels of renin and angiotensinogen, which can induce normal GMCs to produce profibrotic and bioactive substances (da Silva Novaes et al., 2019). Furthermore, HG-induced GMCs can also secrete exosomes with high expression of circ_0125,310 and circ_DLGAP4, which in turn promote the proliferation and fibrosis of GMCs (Bai et al., 2020; Zhu et al., 2022). Furthermore, exosomes derived from GMCs can induce podocyte injury through mechanisms involving miR-4455 or TGF-β1 (Wang et al., 2018; Yu M. et al., 2022). For podocytes, exosomes secreted by these cells under HG conditions can induce TEC damage via miR-221 (Su et al., 2020). For TECs, TEC-derived exosomes promote fibroblast activation through Shh, TGF-β1 mRNA, miR-150, and tumor necrosis factor-α-induced protein 8 (TNFAIP8), which inhibits fibroblast apoptosis and stimulates their proliferation (Liu et al., 2020; Borges et al., 2013; Guan et al., 2020; Liu X. et al., 2023). In addition, HG induces TECs to produce exosomes rich in miR-92a-1-5p, which alters endoplasmic reticulum stress and myoblast transdifferentiation in GMCs (Tsai et al., 2023). M2 macrophage-derived exosomal miR-25-3p can ameliorate HG-induced podocyte injury by activating autophagy (Huang et al., 2020). TECs internalize exosomal miR-155 derived from macrophages, leading to an increase in tubular injury (Zhang Z. et al., 2022). Exosomes secreted by HG-treated macrophages also promote the activation of GMCs by carrying TGF-β1 mRNA or NLRP3 (Zhu Q.-J. et al., 2019; Liu Y. et al., 2023). Furthermore, macrophage-derived exosomes can mediate GEC dysfunction in sepsis-associated acute kidney injury (Xiang et al., 2023).

Exosomes are derived from various types of kidney cells, including glomerular endothelial cells (GECs), mesangial cells (MCs), podocytes, and tubular epithelial cells (TECs). These exosomes encapsulate information-containing biomolecules that are crucial for regulating cell–cell interactions within the renal environment. Furthermore, they play a significant role in modulating pathophysiological processes in the kidneys, such as fibrosis, inflammation, and apoptosis (Figure 2).

GECs represent the primary barrier of the glomerular capillary wall, playing a crucial role in regulating glomerular filtration and constituting an essential component of the inner filtration barrier. Previous studies have reported that GECs treated with high glucose (HG) can secrete increased amounts of exosomes enriched with transforming growth factor-β1 (TGF-β1) mRNA. These GEC-derived exosomes can enhance α-smooth muscle actin expression, promote proliferation, and lead to excessive extracellular matrix protein production in glomerular mesangial cells (GMCs) via the TGF-β1/Smad3 signaling pathway (Wu et al., 2016). Furthermore, HG-treated GECs also release more exosomes containing circular RNAs (circRNAs), which facilitates the intercellular transfer of circRNAs from GECs to GMCs (Ling et al., 2019). Wu et al. demonstrated that GEC-derived exosomes mediate interactions between GECs and podocytes while contributing to the pathogenesis of diabetic nephropathy (DN). In an HG environment, GECs undergo the endothelium-mesenchymal transition (EMT) and produce a significantly higher number of exosomes. These exosomes can mediate the epithelial–mesenchymal transition and dysfunction of podocytes partly through the transfer of TGF-β1 mRNA (Wu et al., 2017).

The MCs of the kidney are specialized stromal cells within the glomeruli that play an important role in maintaining glomerular homeostasis and mediating the glomerular response to injury. Increasing evidence suggests that MCs not only contribute to tissue architecture but also regulate developmental processes, angiogenesis, and cell fate (Avraham et al., 2021). Current findings indicate that exosomes derived from MCs significantly influence the pathophysiology of DN. Exosomes facilitate communication both among MCs themselves and between MCs and podocytes. Research studies have shown that HG-stimulated human mesangial cells (HMCs) release an increased quantity of exosomes containing elevated levels of renin and angiotensinogen. These exosomes can induce the production of pro-fibrotic and biologically active substances in normally cultured HMCs. These results indicate that cellular communication mechanisms mediated by exosomes may play a pivotal role in transmitting and spreading changes to cells that are not directly affected (da Silva Novaes et al., 2019). This pattern of self-action has also been observed in other mechanisms. Exo-circ_0125310, derived from HG-induced MCs, promotes the proliferation and fibrosis of MCs by sponging miR-422a and targeting the IGF1R/p38 axis (Zhu et al., 2022). Similarly, exo-circ_DLGAP4, derived from HG-treated MCs, significantly enhances the proliferation and fibrosis of MCs through the modulation of the miR-143/ERBB3/NF-κB/MMP-2 axis (Bai et al., 2020). Interestingly, recent studies have demonstrated that MC-derived exosomes can play a role in regulating podocyte injury via crosstalk with neighboring cells. Yu M. et al. (2022) found that exosomal miR-4455 from MCs induces podocyte injury in IgA nephropathy by targeting ULK2. Furthermore, it has been reported that exosomes derived from HG-treated MCs can induce podocyte injury through the TGF-β1-PI3K/AKT pathway. Notably, berberine has been shown to protect podocyte function by inhibiting the transfer of TGF-β1 from the GMCs to the podocytes (Wang et al., 2018).

Podocytes are highly differentiated terminal cells that extend from the foot processes, encircling the glomerular basement membrane and maintaining the homeostasis of the glomerular filtration barrier. Increasing evidence suggests that podocyte injury is a critical event in the pathogenesis of DN. Similarly, podocyte-derived exosomes may also contribute to this pathological process. Su et al. demonstrated that podocyte-derived exosomes serve as key mediators of proximal tubular cell injury in diabetes mellitus. Specifically, miR-221 within these exosomes facilitates cellular damage through Wnt/β-catenin signaling pathways. It has been proposed that miR-221 expression induces β-catenin nuclear accumulation by targeting DKK2, an inhibitor of Wnt signaling, ultimately leading to the dedifferentiation of proximal tubular epithelial cells (Su et al., 2020). A recent study found that a significant increase in podocyte-derived exosomes among patients with DN is associated with dysfunctional sirtuin1-mediated lysosomal acidification (Ding et al., 2023). Beyond their effects on proximal tubule cells, podocyte-derived exosomes are implicated in glomerular inflammation. Huang et al. reported that acid sphingomyelins produced by ceramide can participate in regulating NLRP3 inflammasome activation and inflammatory exosome release in podocytes during obesity via the ceramide signaling pathway, resulting in glomerular inflammation and injury associated with obesity-related glomerulopathy (Huang et al., 2023).

TECs are the primary targets of renal injury. Damaged tubular cells release harmful molecules, mediate intercellular communication pathways, and contribute to the progression of renal diseases. Previous studies have demonstrated that exosomes derived from injured TECs can transfer sonic hedgehog (Shh) protein, TGF-β1 mRNA, and miR-150 into fibroblasts, thereby promoting fibroblast activation, proliferation, and stroma production (Borges et al., 2013; Liu et al., 2020). Furthermore, Liu et al. illustrated that tubule-derived exosomes can block p53 signaling by shuttling tumor necrosis factor-α-induced protein 8 (TNFAIP8), which prevents fibroblast apoptosis and stimulates their proliferation (Liu X. et al., 2023). These findings suggest that TEC-derived exosomes have pro-fibrotic functions in kidney diseases. Exosome-mediated delivery plays a crucial role in renal cell crosstalk and the progression of kidney disease. In addition to interacting with fibroblasts, tubule-derived exosomes also engage with mesangial cells. Proximal tubule-derived exosomes contribute to mesangial cell injury in DN via the transfer of miR-92a-1-5p (Tsai et al., 2023).

Exosomes can also be derived from other kidney cell types, including fibroblasts and macrophages. A recent study indicated that exosomes originating from cancer-associated fibroblasts (CAFs) are capable of being internalized by clear cell renal cell carcinoma (ccRCC) cells, which subsequently enhances the proliferation, migration, and invasion of these cancer cells, thereby promoting the progression of ccRCC (Fu et al., 2022). Fibroblasts have been shown to play an important role in the development of renal fibrosis. In their research, Yu et al. observed that TGF-β1 can stimulate the differentiation of renal fibroblasts into myofibroblasts and promote the release of exosomes from these myofibroblasts. Exosomes derived from myofibroblasts enhance the transition of macrophages into myofibroblasts and contribute to renal fibrosis (Yu et al., 2024). Growing evidence indicates that exosomes derived from macrophages are involved in the occurrence and development of kidney diseases through crosstalk with resident renal cells. Studies have shown that miR-25-3p, originating from M2 macrophage-derived exosomes, ameliorates HG-induced podocyte injury by promoting autophagy via the inhibition of DUSP1 expression (Huang et al., 2020). Conversely, exosomes derived from M1 macrophages are known to inhibit podocyte activity and enhance podocyte apoptosis (Liang et al., 2022b). In an ischemia/reperfusion-induced acute kidney injury model, Yuan et al. investigated the crosstalk between macrophages and tubular cells. They discovered that exosomal miRNA-155 released by activated macrophages can be internalized by tubular cells, leading to increased tubule injury by targeting the suppressor of cytokine signaling-1 (Zhang Z. et al., 2022). In an HG environment, it has been observed that macrophage-derived exosomes contain TGF-β1 mRNA, which can trigger mesangial cell activation, proliferation, fibrosis, and inflammation via the TGF-β1/Smad3 pathway (Zhu Q.-J. et al., 2019). In addition, a recent study has reported that macrophage-derived exosomes can promote the activation of the NLRP3 inflammasome and autophagy deficiency of MCs in DN (Liu Y. et al., 2023). Additionally, Xiang et al. (2023) reported that lipopolysaccharides significantly stimulate the secretion of macrophage-derived exosomes. These exosomes have been implicated in inducing dysfunction in GECs.

## 6 Mechanisms of exosomes in renal diseases

### 6.1 Mediating intercellular crosstalk

Exosomes serve as a crucial mechanism for intercellular information transfer, facilitating communication between cells by transporting bioactive molecules such as mRNAs, non-coding RNAs, lipids, and proteins. Their interactions with renal cells are pivotal in the progression of renal diseases. Increasing evidence suggests that exosomes can either exert a local pathogenic effect within nephrons or, through urinary flow, transport pathogenic substances over considerable distances to the distal regions of the nephron, thereby contributing to the pathogenic processes in recipient cells.

In a study utilizing models of LPS-induced acute kidney injury and adriamycin-induced chronic kidney injury, it was observed that the expression of miR-19b-3p was significantly elevated in exosomes derived from TECs compared to control groups. Moreover, this investigation confirmed that TEC-derived exosomes can target the NF-kB/SOCS-1 pathway through a miR-19b-3p-dependent mechanism, leading to the activation of M1 macrophages. This process promotes the progression of tubulointerstitial inflammation and exacerbates renal injury (Lv et al., 2020). Previous research has demonstrated intercellular communication among proximal tubular epithelial cells (PTECs) via exosome transmission in an autocrine pattern. Tsai et al. conducted a study to explore crosstalk within PTECs in DN. They discovered that HG levels stimulate PTECs to upregulate fibronectin-1 (FBLN1), which is subsequently secreted by PTECs through exosomal delivery, thereby inducing the epithelial–mesenchymal transition in these cells (Tsai et al., 2021).

**FIGURE 3:**
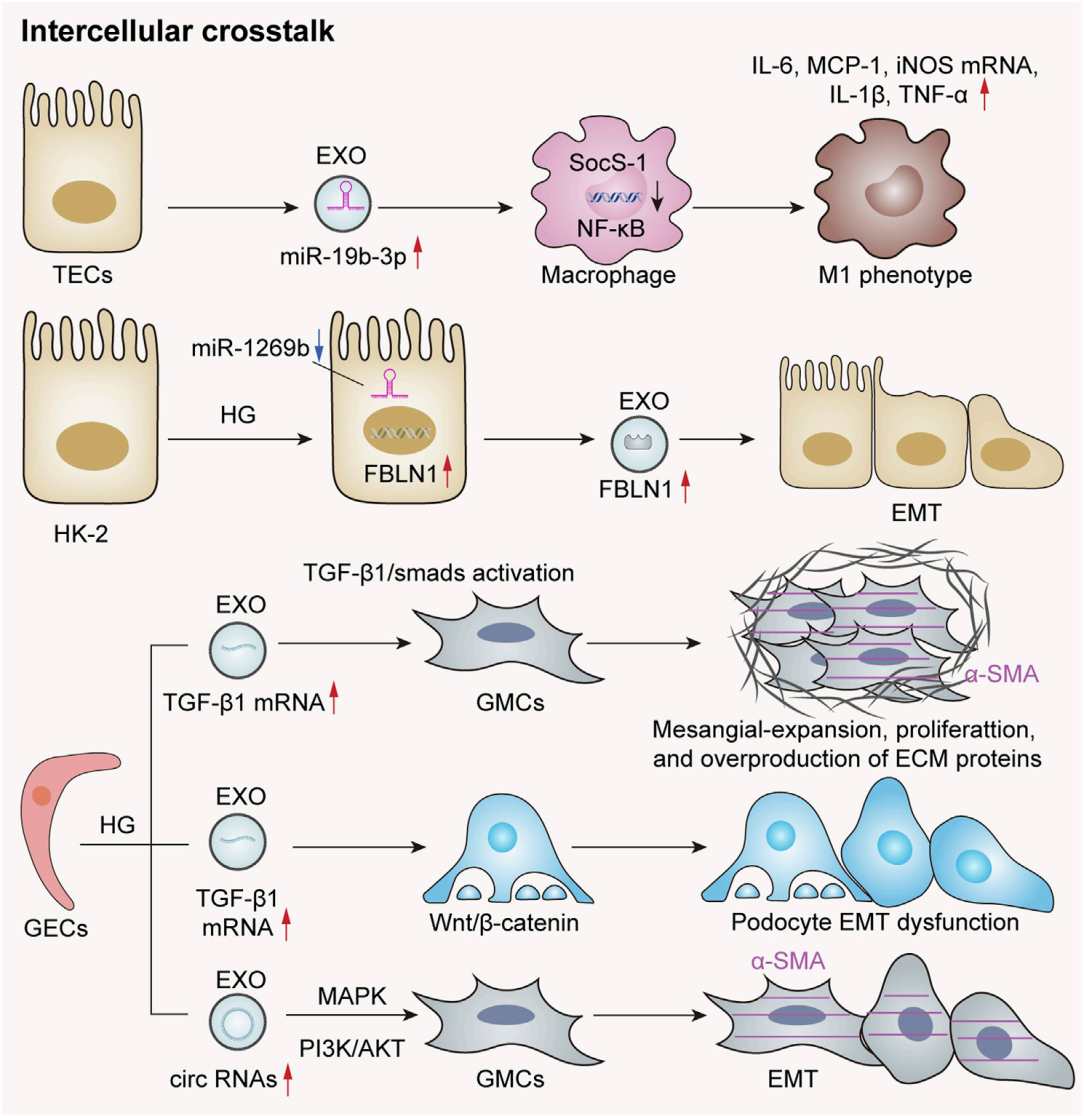
Exosomes mediate crosstalk between kidney cells. The exosomal miR-19b-3p derived from renal TECs is significantly elevated in acute or chronic kidney injury models, leading to M1 phenotypic polarization through internalization by macrophages and targeting the NF-κB/SOCS-1 pathway (Lv et al., 2020). Additionally, the downregulation of miR-1269b in HG-treated HK-2 cells can lead to the increased expression of fibronectin-1 (FBLN1), thereby promoting EMT within these cells (Tsai et al., 2021). HG-induced GECs secrete exosomes rich in TGF-β1 mRNA, which are subsequently internalized by GMCs and mediate GMC activation via the TGF-β1/Smad signaling pathway. Furthermore, exosomes abundant in circRNAs are released to enhance α-smooth muscle actin (α-SMA) expression in GMCs and induce the EMT. Notably, HG-treated GECs also release exosomes rich in TGF-β1 mRNA, which contribute to EMT and podocyte dysfunction through the activation of the Wnt/β-catenin signaling pathway (Hu et al., 2024).

Cells within the glomeruli primarily comprise GECs, podocytes, and mesangial cells. These cell types are capable of releasing exosomes, facilitating crosstalk between different cell populations through these exosomal mediators. This interaction plays a pivotal role in the pathogenesis of glomerular and renal tubular diseases (Hu et al., 2024). Tubulointerstitial fibrosis is a hallmark characteristic of progressive chronic kidney disease. However, its development necessitates the involvement of various cell types—including endothelial cells, fibroblasts, and inflammatory cells—within the tubular interstitium. Following severe injury to renal tubular epithelial cells, exosomes containing diverse active substances can be released. These exosomes function analogously to virus-like seeds, thereby leading to fibroblast activation, inflammatory cell recruitment, and endothelial cells (Guo et al., 2023). Moreover, there exists significant signal crosstalk between tubular epithelial cells and interstitial fibroblasts following renal injury. This interaction contributes to the progression of tubular interstitial damage (Tan et al., 2016). In summary, such crosstalk among different nephron components is exceedingly prevalent in instances of kidney injury (Figure 3).

### 6.2 Inflammation and immune regulation

**FIGURE 4:**
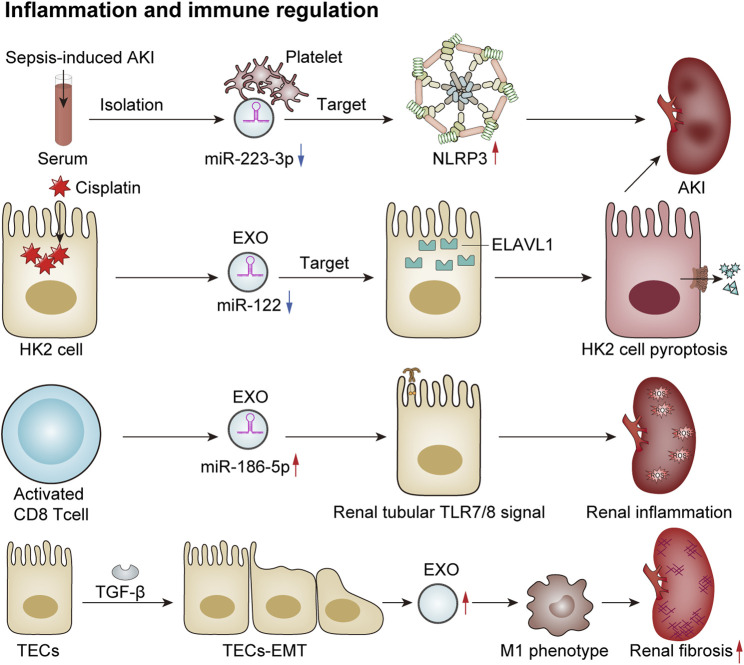
Exosomes mediate immune regulation and renal inflammation. In a sepsis-induced mouse model of AKI, reduced levels of platelet-derived exosomal miR-2233p inhibit endothelial cell pyroptosis, thereby exacerbating AKI by targeting NLRP3 (Wan et al., 2024). Additionally, in cisplatin-treated HK2 cells, the expression of exosomal miR-122 is downregulated, which promotes cellular pyroptosis and contributes to AKI by targeting embryonic lethal abnormal vision (ELAVL1) (Zhu et al., 2023). Activated CD8 T cells release exosomes enriched with elevated levels of miR-186-5p, which activate the renal tubular TLR7/8 signal axis to trigger renal inflammation and tissue damage (Xu et al., 2023). Furthermore, TGF-β induces TECs to undergo the EMT and secrete more exosomes, which induces macrophage M1 polarization and promotes the progression of renal fibrosis (Lu et al., 2023).

Exosomes play an important role in inflammation and immune regulation in renal diseases. They can regulate the intensity and course of the immune response by modulating the release of inflammatory factors and the activity of inflammatory cells, thereby impacting both the progression and regression of renal diseases. An increasing number of studies have shown that exosomes can directly or indirectly regulate the immune-inflammatory response through miRNAs, thus participating in kidney injury. In a model of sepsis-induced acute renal injury, Wan et al. observed a significant downregulation in levels of miR-223-3p derived from platelet exosomes. This alteration led to the activation of the NLRP3 inflammasome and pyroptosis in endothelial cells, consequently exacerbating the inflammatory response associated with sepsis-induced acute kidney injury (Wan et al., 2024). Additionally, another study reported that exosomes derived from HK2 cells influenced cisplatin-induced acute kidney injury and HK2 cell pyroptosis via the miR-122/embryonic lethal abnormal vision (ELAVL1) axis (Zhu et al., 2023). A recent study has proposed that activated CD8^+^ T cells contribute to renal inflammation and tissue injury by releasing exosomes enriched with miR-186-5p. Furthermore, miR-186-5p is suggested to initiate tubular cell apoptosis through the direct activation of the TLR7/8 signaling axis in renal tubules (Xu et al., 2023). Macrophages play a crucial role as inflammatory cells within the kidney, and their functions are influenced by their phenotypic states. Kidney-derived exosomes can modulate the macrophage phenotype via crosstalk, thereby participating in the inflammatory processes associated with kidney disease. Exosomes released from tubular epithelial cells undergoing EMT due to TGF-β treatment have been shown to induce M1 macrophage activation, which may lead to the progression of renal fibrosis (Lu et al., 2023). In conclusion, exosomes are involved in various renal diseases by regulating inflammatory responses and immunological activities (Figure 4).

### 6.3 Cell regeneration, repair, and fibrosis

**FIGURE 5:**
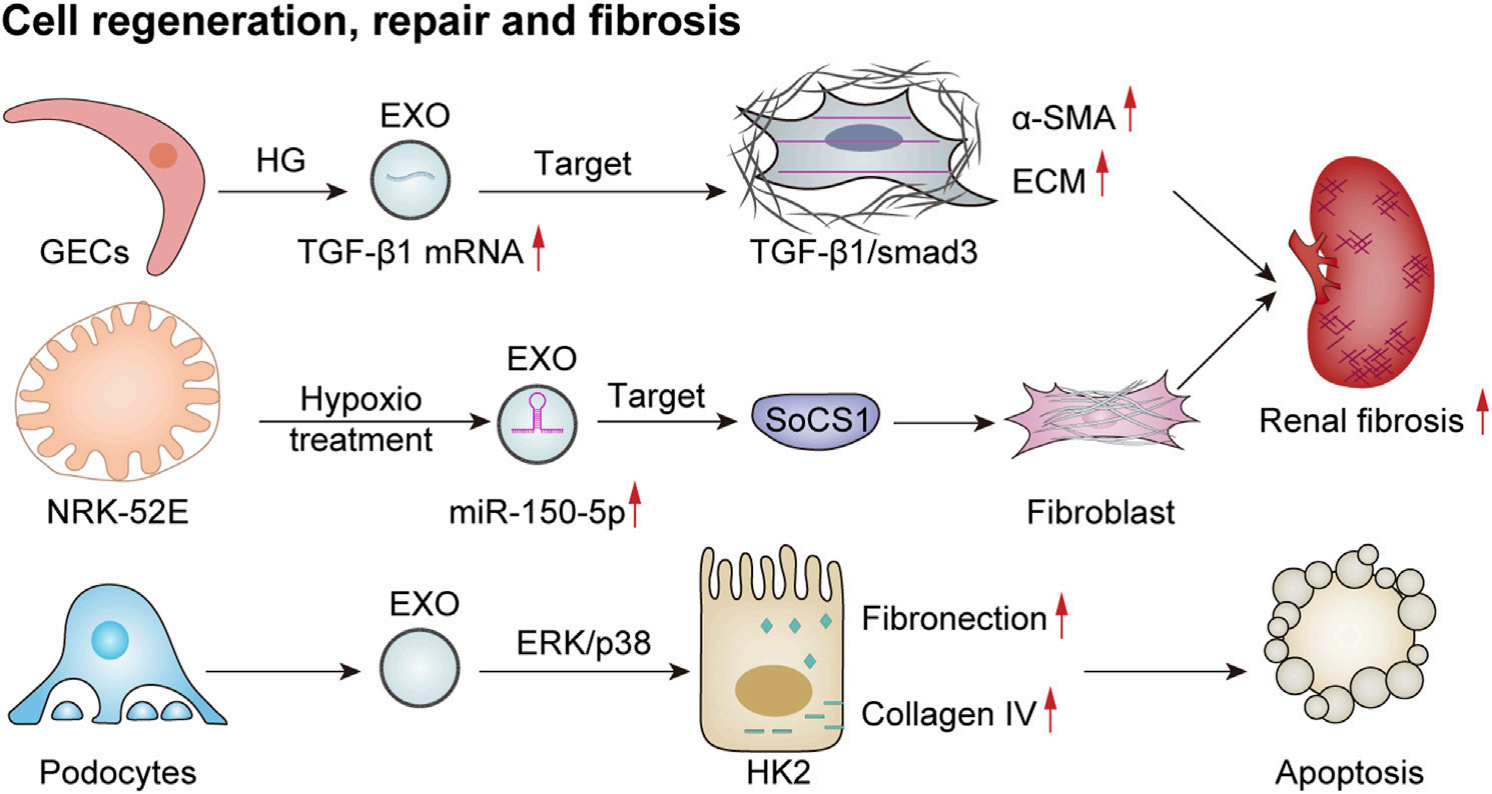
Exosomes mediate cell regeneration, repair, and fibrosis. HG-treated GECs secrete more TGF-β1 mRNA-rich exosomes, which enhance the expression of α-smooth muscle actin (α-SMA), promote proliferation, and lead to excessive production of extracellular matrix proteins (ECMs) in GMCs via the TGF-β1/Smad3 signaling pathway, ultimately resulting in renal fibrosis (Wu et al., 2016). EVs derived from damaged podocytes can activate the ERK and p38 pathways in HK2 cells, thereby increasing fibronectin and collagen IV expression and promoting apoptosis (Jeon et al., 2020). Additionally, hypoxia-treated renal tubular epithelial cells (NRK-52E) release exosomes with increased miR-150-5p expression, thus targeting cytokine signaling 1 (SOCS1) to promote fibroblast activation and exacerbate renal fibrosis (Zhou et al., 2021).

Exosomes have been proposed to play a significant role in promoting repair and regeneration in renal diseases. However, under certain conditions, they may also be involved in the development of fibrosis within the kidney, thereby exacerbating interstitial fibrosis (Figure 5). Renal fibrosis represents a major pathological change observed in the chronic kidney, ultimately progressing to end-stage renal failure. This condition is characterized by the substantial accumulation of the extracellular matrix within the renal interstitium, destruction of renal tissue architecture, and subsequent loss of function. Emerging evidence indicates that following kidney injury, exosomes derived from glomerular endothelial cells, podocytes, renal tubular epithelial cells, and other resident cell types recruit inflammatory cells through various cytokines and pathways. These processes activate myofibroblasts and lead to excessive deposition of the extracellular matrix components, thus promoting the progression of renal fibrosis.

Previous studies have shown that exosomes derived from HG-treated glomerular endothelial cells activate mesangial cells, thereby promoting renal fibrosis (Wu et al., 2016). In addition, a study involving the culture of renal tubular epithelial (HK2) cells with exosomes obtained from either puromycin-treated or healthy human podocytes revealed that exosomes from damaged podocytes significantly increased the expression of fibronectin and collagen IV in renal tubular epithelial cells. This effect was mediated through the activation of ERK and P38 signaling pathways, contributing to tubular epithelial cell injury associated with tubulointerstitial fibrosis in renal disease (Jeon et al., 2020). In a model of acute renal injury induced by ischemia–reperfusion, there was a notable increase in exosomes derived from renal tubular epithelial cells. These exosomes were found to negatively regulate the expression of suppressor of cytokine signaling 1 via miR-150-5p, which subsequently activated fibroblasts and facilitated the progression of renal fibrosis (Zhou et al., 2021). This body of evidence suggests a potential association between exosomes and renal fibrosis. However, further in-depth studies are necessary to elucidate the specific mechanisms underlying this relationship.

### 6.4 Tumor growth and metastasis

**FIGURE 6:**
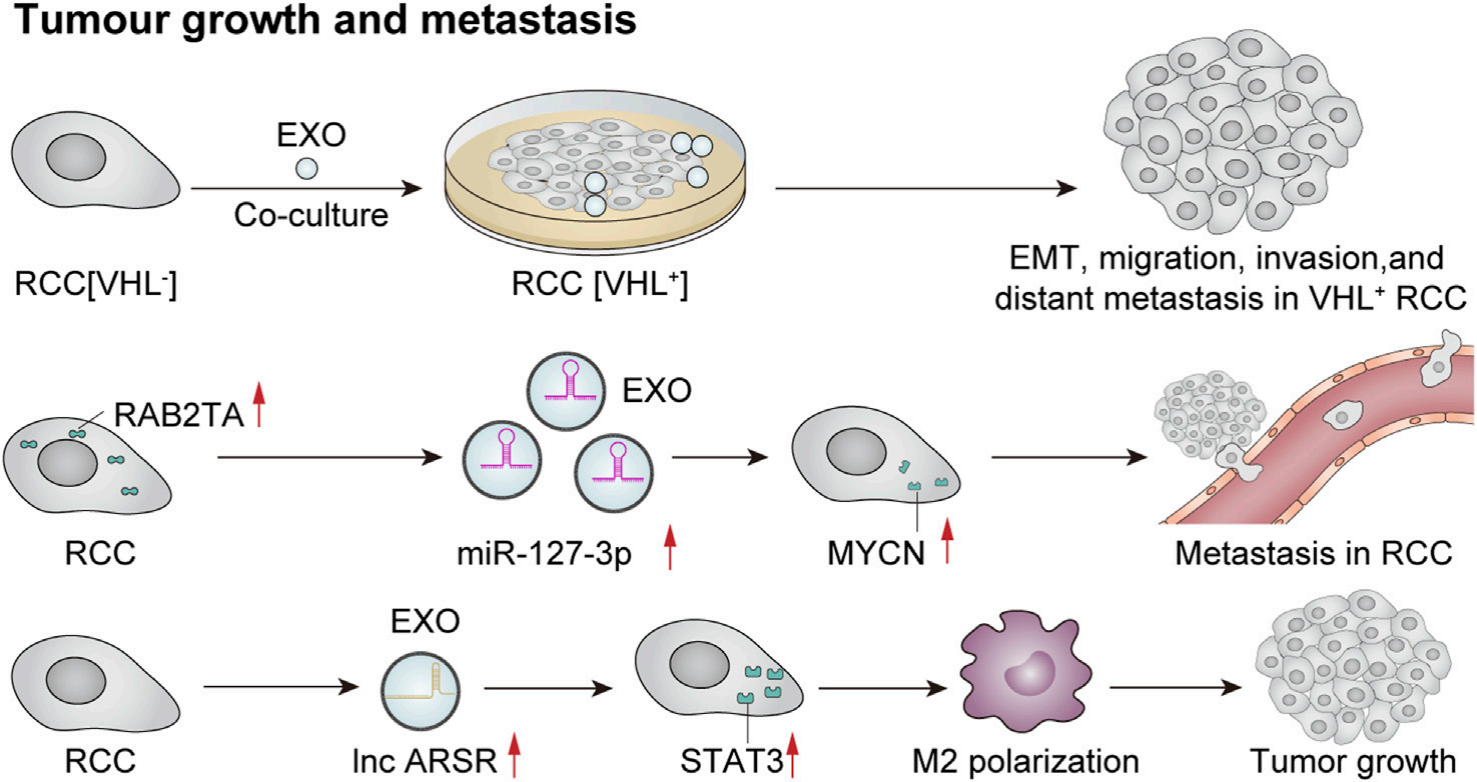
Exosomes mediate tumor growth and metastasis. Co-culture of VHL-renal cell carcinoma (RCC)-derived exosomes with VHL + RCC cells can promote EMT, migration, invasion, and distant metastasis of VHL + cells (Flora et al., 2023). Furthermore, RCC cells that overexpress RAB27A secrete a higher quantity of miR-127-3p-rich exosomes, resulting in elevated MYCN protein levels and augmented metastatic potential of cancer cells (Song et al., 2024). Additionally, RCC-derived exosomes contain significant amounts of lncRNA ARSR, which elevate the expression of signal transducer and activator of transcription 3 (STAT3), promote M2 polarization of macrophages, and contribute to tumor growth (Zhang et al., 2022b).

Recently, several studies have indicated that exosomes play a crucial role in tumor growth and metastasis through mechanisms such as intercellular communication, microenvironmental regulation, and immune evasion. Flora et al. observed a loss of function of the von Hippel–Lindau (VHL) oncogene in 70% of clear cell renal cell carcinoma (ccRCC) cases, which is considered an early oncogenic driver of this malignancy. VHL (−) cells can produce significantly higher levels of exosomes. Moreover, exosomes derived from VHL (−) RCC cells can induce epithelial–mesenchymal transition, migration, invasion, and distant metastasis in VHL (+) RCC cells upon uptake (Flora et al., 2023). In addition, Song et al. (2024) reported that exosome-mediated secretion of miR-127-3p can accelerate metastasis in renal cell carcinoma. A recent study investigated the role of tumor-derived exosomes in macrophage phenotypic transformation and tumor development in renal cell carcinoma (RCC). The findings revealed that RCC-derived exosomes carry elevated levels of lncARSR, activate the STAT3 pathway, induce a shift in the macrophage phenotype from M1 to M2, and promote cytokine secretion along with phagocytosis and angiogenesis—thereby significantly enhancing tumor progression (Zhang et al., 2022b). Exosomes influence tumor development through intercellular communication, microenvironmental regulation, and immune system interactions. An extensive exploration of these mechanisms may offer new avenues for future cancer treatments and facilitate the development of therapeutic strategies targeting exosomes to prevent or mitigate tumor growth or proliferation (Figure 6).

## 7 Application of exosomes in kidney diseases

Urine is an ideal material for evaluating kidney disease. Variations in the quantity, origin, or composition of exosomes isolated from urine are closely associated with alterations in the physiological and pathological states of the kidney. Therefore, exosomes can be used as a non-invasive diagnostic tool, showing great potential for application in the early diagnosis, prognostic assessment, and monitoring of kidney diseases (Pisitkun et al., 2004). Moreover, relevant studies indicate that exosomes may function as efficient drug carriers due to their natural substance transport properties, inherent long-term recycling capabilities, and superior biocompatibility. They also hold promise as a gene therapy tool, providing innovative approaches for developing new therapeutic strategies across various diseases (Batrakova and Kim, 2016). Hence, there has been a growing body of studies focused on applying exosomes in diagnosing and treating kidney diseases.

### 7.1 IgA nephropathy

IgA nephropathy (IgAN) is an autoimmune glomerular disease characterized by a wide spectrum of clinical presentations, ranging from asymptomatic microscopic hematuria to acute progressive nephritis. Therefore, non-invasive biomarkers that reflect histological changes are essential for predicting the progression of IgA nephropathy and determining therapeutic strategies (Feng et al., 2018). Exosomes have emerged as promising novel non-invasive biomarkers for both the diagnosis and treatment of IgAN. Current evidence indicates that the diagnostic biomarkers associated with IgAN primarily originate from urinary exosomal nucleic acids, while other components of exosomes are rarely reported.

Previous studies have explored the differences in urinary exosomal miRNA expression profiles between IgAN patients and healthy controls, revealing significant disparities in these profiles. Among the identified exosomal miRNAs, miR-29c, miR-146a, and miR-205 have emerged as potential novel non-invasive biomarkers for IgAN (Min et al., 2018). Additionally, hsa-miR-451a, hsa-let-7d-3p, and miR-199a-3p may also be used as non-invasive indicators for evaluating IgAN (Li S. et al., 2022). The findings presented by Zhao et al. are particularly compelling. Their results showed that the expression of miR-4639 and miR-210 was markedly upregulated in patients with IgAN. Moreover, increased expression of miR-4639 was associated with more severe histological activity. Importantly, follow-up assessments revealed that exosomal levels of both miR-4639 and miR-210 outperformed proteinuria measurements in predicting renal prognosis. This suggests that these markers may be effective tools for diagnosing IgAN, assessing disease severity, and monitoring progression (Zhao S. et al., 2022). In addition to miRNAs, exosomal lncRNAs and mRNA may also serve as diagnostic biomarkers for IgAN. One study identified that lncRNA-G21551 was significantly downregulated in patients with IgAN when comparing their plasma exosomes to those of their healthy first-degree relatives. The downregulation of lncRNA-G21551 suggests its potential utility as a non-invasive biomarker for IgAN (Guo et al., 2020). In another study, Feng et al. discovered that urinary exosomal chemokine (C-C motif) ligand 2 (CCL2) levels were correlated with the glomerular filtration rate, tubulointerstitial inflammation, and the progression of renal dysfunction. Consequently, urinary exosomal CCL2 mRNA emerges as a promising biomarker for monitoring renal tissue injury and the deterioration of renal function in IgAN (Feng et al., 2018).

Previous reports have highlighted the application of exosomes in the treatment of IgAN. An *in vitro* study demonstrated that the combination of artemisinin and hydroxychloroquine (AH) enhanced exosome secretion from human renal tubular epithelial cells (HK-2), effectively inhibiting NF-κB/NLRP3 signaling activation in human mesangial cells. This inhibition subsequently reduced pro-inflammatory cytokine production, thereby providing a novel therapeutic strategy for IgAN (Bai et al., 2019). Recent studies have focused on the role of exosomes in the therapeutic response of patients with IgAN. Wu J. et al. (2022) found that plasma exosomal IRAK1 could serve as a potential biomarker for predicting the treatment response to renin–angiotensin system inhibitors among individuals with IgAN. Additionally, another study revealed that urinary exosomal miR-451a may serve as a promising biomarker for diagnosing IgAN and assessing tubulointerstitial damage. Further follow-up studies indicated that the baseline levels of urinary exosomal miR-451a may predict both therapeutic efficacy and disease progression in IgAN patients (Zhang Q. et al., 2024).

### 7.2 Membranous nephropathy

Membranous nephropathy accounts for approximately 20% of primary nephrotic syndrome in China, typically presenting with an insidious onset. Renal function deterioration generally manifests gradually over a period of 5–10 years following the initial onset of the disease. As the condition progresses, pathological changes intensify, and treatment efficacy diminishes. Therefore, early diagnosis and timely intervention are essential to prevent further decline in renal function. By comparing the expression profiles of exosomes found in the serum and urine of patients with idiopathic membranous nephropathy (IMN) against those of normal controls using gene sequencing technology, Ma et al. (2019) identified significant differences in specific circRNA expressions within the exosomes from IMN patients. Among these, MUC3A can be considered a potential diagnostic biomarker for IMN. In addition, one study revealed that urinary exosomal hsa_circ_0001250 could serve as another promising biomarker for IMN (Li Q. et al., 2022). Subsequently, more urinary exosomal miRNAs specifically expressed in patients have been screened through high-throughput sequencing, and some of them showed increased expression levels, whereas others showed decreased expression levels. Despite their strong association with kidney disease, their potential value as diagnostic biomarkers for IMN remains unclear and warrants further investigation (Zhang J. et al., 2020). Evidence suggests that miR-30b-5p and miR-9-5p may represent novel non-invasive biomarkers for IMN (Guo et al., 2022). In addition to nucleic acids utilized as biomarkers for diagnosing IMN, proteins in urinary exosomes may also have a significant diagnostic value. A recent study revealed that urinary exosomal PLA2R can effectively diagnose PLA2R-associated membranous nephropathy with a sensitivity of 95.4% and specificity of 63.3%. Furthermore, the combined assessment of urinary exosomal PLA2R and the serum anti-PLA2R antibody demonstrates enhanced sensitivity (Wang B. et al., 2024).

### 7.3 Focal segmental glomerulonephritis

Focal segmental glomerulosclerosis (FSGS) is a prevalent primary glomerular disease, primarily affecting children and serving as a common cause of nephrotic syndrome in this population. FSGS is characterized by lesions involving partial glomerular and/or capillary loops. Earlier studies have shown that urinary exosomal Wilms’ tumor-1 (WT-1) is a promising non-invasive biomarker with significant podocyte specificity, reflecting early podocyte injury in patients with FSGS (Zhou H. et al., 2013). In a recent study, it was found that urinary exosomal miR-193a levels were significantly elevated in patients diagnosed with primary FSGS compared to those with minimal change nephropathy and IgAN. Furthermore, these levels exhibited a positive correlation with the glomerulosclerosis index. These findings suggest that urinary exosomal miR-193a can be used as a reliable non-invasive biomarker for facilitating early diagnosis and prognostic evaluation in individuals suffering from FSGS (Wang et al., 2021). Similar results were corroborated by another study, which reported an area under the ROC curve of 0.85 for urinary exosomal miR-193a in diagnosing primary FSGS (Huang et al., 2017). In addition to miR-193a, one study analyzed the expression profiles of urine and plasma exosomal microRNAs in patients presenting minimal change disease (MCD) versus those with FSGS. The findings revealed that compared to both MCD and control groups, the levels of miR-1915 and miR-663 were downregulated in the urinary exosomes of patients with FSGS, whereas the level of miR-155 was upregulated (Ramezani et al., 2015).

### 7.4 Diabetic nephropathy

Diabetic nephropathy (DN), also known as diabetic kidney disease (DKD), is one of the most common microvascular complications in diabetes mellitus (DM) patients. Currently, it is the primary cause of chronic kidney disease (CKD) and ESRD (Sagoo and Gnudi, 2020). Microalbuminuria is a widely utilized assessment tool for DKD. Clinically, microalbuminuria, urinary albumin-to-creatinine ratio (ACR), and/or serum creatinine-based estimated glomerular filtration rate (eGFR) are commonly used to assess glomerular injury and renal function. These parameters are also frequently used in the assessment of DN. However, in the early stages of DN, reliance on urinary microalbumin levels and eGFR is less reliable and sensitive for early diagnosis. Kidney biopsy is regarded as the gold standard for diagnosing DN. However, it is an invasive procedure that requires highly experienced technical operators. Furthermore, renal biopsy is not routinely performed in all patients with DN but is recommended primarily when nondiabetic nephropathy is suspected. Therefore, it is important to identify promising biomarkers and develop effective therapeutic agents to prevent, diagnose, and treat DN. Developing biomarkers for the early diagnosis of DN has become a hot topic in nephropathy research. Recent studies have increasingly demonstrated that nucleic acids and proteins found within urinary exosomes can be used as biomarkers for both the diagnosis and prognosis of DN (Table 2).

**TABLE 2:** Potential biomarkers in exosomes of DN.

Potential biomarker		Subject	Source of exosomes	Detection method	Main findings	Reference
miRNAs						
	miR-451-5p	Diabetic rats	Urinary exosomes	Sequencing technology and qPCR validation	It was significantly increased in DM rats during the progression of diabetes and was better than urinary albumin excretion in predicting kidney damage in DM.	Mohan et al. (2016)
	let-7c-5p	T2DM/DN patients and HCs	Urinary exosomes	RT-PCR	The upregulated expression of urinary exosomal let-7c-5p is associated with glomerular filtration rate and disease progression.	Li et al. (2018)
	miR-15b, miR-34a, and miR-636	T2DM patients	Urinary exosomes	Bioinformatic analysis, PCR array analysis, and qPCR validation	There were significant correlations among the three miRNAs, which share common target genes related to glucose homeostasis, angiogenesis, and DKD.	Eissa et al. (2016)
	miR-21–5p and miR-30b-5p	T2DM/DKD patients	Urinary exosomes	PCR panels	Increased expression of urinary exosomal miR-21-5p and decreased expression of miR-30b-5p can better distinguish individuals with impaired renal function, but there is no specific association with DKD.	Zang et al. (2019)
	miR-4534	T2DM/DKD patients	Urinary exosomes	miRNA microarray analysis and qRT-PCR validation	It has a characteristic elevated expression in DKD and is strongly correlated with microproteinuria.	Zhao et al. (2020)
	miR-145-5p and miR-27a-3p	T2DM/DKD patients and HCs	Urinary exosomes	RT-qPCR and bioinformatics analysis	They are significantly upregulated in DKD and associated with the progression of kidney injury.	Han et al. (2024)
	miR-615–3p	T2DM/DKD patients and HCs	Urinary exosomes	RT-qPCR validation	The expression level in DKD patients was significantly elevated. It was positively or negatively correlated with some clinical indicators of DKD. The combination with ACR has a more stable and sensitive diagnostic criteria.	Wang et al. (2023a)
mRNAs	WT1	DN/MCNS patients and HCs	Urinary exosomes	RT-qPCR	WT1 mRNA levels reflect glomerular damage in diabetic patients and predict a future decline in eGFR.	Abe et al. (2018)
	CCL21	DN/DM patients, HCs, and DN rat	Urinary EVs	RT-PCR	The increase in CCL21 mRNA in urine small EVs in DN patients can more effectively distinguish early DN patients from DM patients.	Feng et al. (2021)
	WT1 and ACE	DN/DM patients and HCs	Blood EVs	qRT-PCR	The expression of WT1 mRNA in the blood EVs of DN patients was significantly increased, and the accuracy of predicting early DN was 0.63. On the contrary, ACE mRNA expression was significantly decreased, and the accuracy of predicting early DN was 0.62.	Hashemi et al. (2021)
	CDH2 and MCP-1	DN/DM patients and HCs	Blood EVs	qRT-PCR	CDH2 and MCP-1 mRNA expressions in blood EVs were significantly downregulated in DN, and both were inversely correlated with creatinine and the Alb/Cr ratio.	Dehghanbanadaki et al. (2022)
Proteins	WT1	Type-1 DM patients and HCs	Urinary exosomes	Western blotting	The predominant expression of the WT1 protein in urinary exosomes in type-1 diabetic patients is closely related to increased urinary protein excretion and decreased eGFR.	Kalani et al. (2013)
	PAK6 and EGFR	DN/T2DM patients and HCs	Urinary exosomes	LC–MS/MS; Western blot and ELISA validation	PAK6 and EGFR were significantly upregulated in DN, negatively correlated with eGFR, and positively correlated with serum creatinine levels. The AUC value of PAK6 diagnostic DN was 0.903, EGFR was 0.842, and the combination of the two proteins was 0.912.	Li et al. (2023b)
	CALM1	DN/T2DM patients and HCs	Urinary exosomes	LC–MS/MS; Western blot and ELISA validation	CALM1 correlated with serum Cr and eGFR levels, and a diagnostic model based on CALM1 and serum ALB levels was established.	Li et al. (2023a)
	TF, SERPINA1, AFM, and CTSD	DN/T2DM patients and HCs	Urinary exosomes	LC–MS/MS detection; Western blot validation	A large-scale urinary exosomal proteomic analysis was performed on T2DM patients without or with DKD in different stages, and it was found that TF, SERPINA1, and AFM expressions increased with the progression of DKD, while CTSD decreased.	Du et al. (2023)

DN, diabetic nephropathy; qPCR, quantitative real-time polymerase chain reaction; DM, diabetes mellitus; T2DM, type 2 diabetes mellitus; DKD, diabetic kidney disease; HCs, healthy controls; RT-PCR, real-time quantitative polymerase chain reaction; ACR, albumin-to-creatinine ratio; WT1, Wilms tumor 1; eGFR, estimated glomerular filtration rate; EVs, extracellular vesicles; CDH2, cadherin 2; MCP-1, monocyte chemoattractant protein-1; PAK6, serine/threonine-protein kinase PAK6; EGFR, epidermal growth factor receptor; AUC, area under curve; CALM1, calmodulin-1; TF, transferrin; SERPINA1, Serpin α1-antitrypsin; AFM, afamin; CTSD, cathepsin D.

Recently, numerous studies have focused on the therapeutic potential of exosomes in various diseases. Studies have demonstrated that exosomes derived from different stem cell types have significant potential for treating DN. Previous investigations have established that MSCs are multifunctional stem cells located in adipose tissue, bone marrow, umbilical cord, placenta, and other tissues and organs. MSCs display remarkable self-renewal capabilities and differentiation potential. An increasing number of studies have reported that MSC-derived exosomes—particularly those sourced from adipose tissue, bone marrow, and umbilical cord—can exert protective effects on renal function. The underlying mechanisms primarily involve enhancing renal function and mitigating pathological damage to the kidney by reducing inflammation and inhibiting fibrosis and oxidative stress while promoting renal tissue repair (Table 3).

**TABLE 3:** Potential therapeutic values of exosomes derived from different cell types in DN.

Source of exosomes	Cell or animal model	Key factor	Mechanism	Reference
ADSCs	Diabetic mice and MPC5 podocytes	miR-486	The expression of miR-486 was enhanced, and activating the Smad1/mTOR signaling pathway in podocytes was inhibited, leading to increased autophagy and reduced podocyte apoptosis	Jin et al. (2019)
	MPC5 podocytes	miR-215–5p	By inhibiting ZEB2 transcription, miR-215–5p is mediated to shuttle into podocytes, inhibiting HG-induced EMT progression and podocyte migration	Jin et al. (2020)
	Diabetic mice and MP5 podocytes	miR-26a-5p	miR-26a-5p is delivered to podocytes and alleviates pathological symptoms of DN by mediating TLR4/NF-κB/VEGFA signaling pathway inactivation	Duan et al. (2020)
	Diabetic mice and MPC5 podocytes	Nrf2/Keap1 pathway and FAM129B	Regulating the Nrf2/Keap1 pathway to alleviate inflammation and oxidative stress in DN by targeting FAM129B	Ren et al. (2023)
	GMC and DN rats	miR-125a	Carried miR-125a can be targeted to bind HDAC1 and downregulate ET-1, thereby inhibiting mesangial hyperplasia and renal fibrosis, promoting cell apoptosis, inhibiting DN progression, and relieving symptoms	Hao et al. (2021)
	MPC5 cells	miR-15b-5p	miR-15b-5p from ADSC-derived EVs can inhibit VEGF expression by binding to PDK4, thereby reducing MPC5 cell apoptosis and inflammation	Zhao et al. (2022b)
BMSCs	STZ-diabetic mice	Fibrotic markers (collagen I, TGF-β, and α-SMA) and fibrosis-related genes	BMSCs–EVs can ameliorate renal dysfunction, attenuate renal histopathological changes, and reduce fibrosis	Grange et al. (2019)
	DN rats	Autophagy mTOR pathway	Renal function is improved, and renal tissue histology is restored. LC3 and Beclin-1 are significantly increased, and the expressions of mTOR and fibrosis markers in renal tissue are significantly decreased	Ebrahim et al. (2018)
	HKCs	miR-125b	BMSC-derived exosomal miR-125b can induce autophagy and inhibit apoptosis in HG-treated HKCs *via* the TRAF6/AKT axis	Cai et al. (2021)
	HK-2 cells	miR-30e-5p	BMSC-derived exosomal miR-30e-5p can target ELAVL1 and inhibit caspase-1-mediated pyroptosis	Lv et al. (2023)
	Diabetic rats	Apoptosis-associated proteins and inflammatory molecules TNF-α and NF-κB	Inhibiting apoptosis and inflammation	Liu et al. (2023a)
USCs	Diabetes rat	Molecules associated with apoptosis, angiogenesis, and cell survival	Inhibiting podocyte apoptosis and promoting angiogenesis and cell survival	Jiang et al. (2016)
	Diabetic rats and HPDCs	miR-16–5p	miR-16-5p can inhibit VEGFA expression and podocyte apoptosis, thereby ameliorating HG-induced podocyte injury	Duan et al. (2021)
	DN rats and macrophage RAW264.7	circRNA ATG7	Promoting macrophage M2 polarization by regulating the SOCS1/STAT3 signaling pathway through miR-4500	Sun et al. (2024)
M2 macrophage	Macrophage RAW264.7	miR-25-3p	miR-25-3p activates autophagy *via* inhibiting DUSP1 expression and improves podocyte injury induced by high glucose	Huang et al. (2020)
hUC-MSCs	Diabetic mice and podocytes	miR-22-3p	miR-22-3p may reduce the NLRP3 signaling pathway by inhibiting the expression of NLRP3 and alleviating the NLPR3-mediated inflammatory response	Wang et al. (2023c)
	DN rats and TECs	Hedgehog/SMO signaling molecules	Inhibiting the Hedgehog/SMO pathway reduces tubular epithelial EMT and alleviates renal fibrosis	Zhang et al. (2024a)
	DN rats and macrophage RAW264.7	miR-146a-5p	miR-146a-5p can alleviate renal damage in DN rats by facilitating M2 macrophage polarization by targeting TRAF6	Zhang et al. (2022c)
	Db/db mice and HK2 cells	miR-424-5p	Inhibiting HG-induced apoptosis and EMT through miR-424–5p targeting of YAP1	Cui et al. (2022)
Skeletal muscle satellite cells	DN patients, db/db mice, and TECs	miR-23a/27a/26a	Ameliorating renal tubular injury and the progression of TIF in DN mice	Ji et al. (2023)

ADSCs, adipose-derived mesenchymal stem cells; DN, diabetic nephropathy; MPC5, mouse podocyte clone 5; HG, high glucose; EMT, endothelium-mesenchymal transition; ZEB2, zinc finger E-box-binding homeobox 2; TLR4, toll-like receptor 4; VEGFA, vascular endothelial growth factor A; BMSCs, bone marrow mesenchymal stem cells (BMSCs); Nrf2, nuclear factor erythroid 2-related factor 2; Keap1; kelch-like ECH-associated protein 1; GMC, glomerular mesangial cell; HDAC1, histone deacetylase 1; ET-1, endothelin-1; HKCs, human embryonic kidney epithelial cells; TRAF6, tumor necrosis factor receptor-associated factor 6; ELAVL1, ELAV, like RNA, binding protein 1; USCs, urine-derived stem cells; HPDCs, human podocytes; ATG7, autophagy-associated gene 7; DUSP1, dual specificity protein phosphatase 1; hUC-MSCs, human umbilical cord mesenchymal stem cells; SMO, smoothened; TECs, tubular epithelial cells; YAP1, Yes-associated protein 1; TIF, tubulointerstitial fibrosis.

### 7.5 Lupus nephritis

Lupus nephritis (LN) represents a severe manifestation of systemic lupus erythematosus (SLE). The hallmark pathological feature of LN is the deposition of an immune complex formed by the combination of autoantibodies and antigens in the glomeruli. Early diagnosis of renal disease in SLE patients is crucial. Currently, renal biopsy is regarded as the gold standard for diagnosing LN. However, it is invasive. Therefore, searching for a non-invasive diagnostic marker is essential. Recently, numerous studies have demonstrated that specific components of urine- or blood-derived exosomes can be used as biomarkers for the early diagnosis of LN and for monitoring the therapeutic response. Among these components, miRNAs have been extensively studied. Growing evidence from both clinical and experimental models suggests that miRNAs and tRNA-derived small non-coding RNAs (tsRNAs) play pivotal roles in the progression of LN (Table 4).

**TABLE 4:** Potential biomarkers in exosomes of LN.

Potential biomarker		Subject	Source of exosomes	Detection method	Main finding	Reference
miRNA						
	miR-146a	SLE with or without patients with LN and HCs	Urinary exosomes	RT-PCR	All urinary miRNAs tested were mainly in exosomes in patients with LN. Only miR-146a can discriminate the presence of active LN	Perez-Hernandez et al. (2015)
	miR-146a	Patients with SLE, patients with LN, and HCs	Urinary exosomes	RT-qPCR	miR-146a levels are correlated with lupus activity, proteinuria, and histological features	Perez-Hernandez et al. (2021)
	miR-29c	Patients with LN and non-lupus chronic kidney diseases and HCs	Urinary exosomes	RT-PCR	miR-29c levels decreased significantly in patients with LN but lacked specificity. It can predict the degree of chronicity and correlate negatively with Smad3 and MMP2 expression	Solé et al. (2015)
	miR-21, miR-150, and miR-29c	Patients with LN and HCs; RMCs and RTCs	Urinary exosomes	RT-qPCR	The combined use of three miRNAs provides a non-invasive method for early renal fibrosis and the prediction of disease progression in patients with LN.	Solé et al. (2019)
	miR-195-5p	SLE with or without patients with LN	Urinary exosomes	Bioinformatic screen and RT-qPCR	The expression of miR-195-5p is decreased in patients with SLE, and it can distinguish LN from SLE	Cheng et al. (2022)
	miR-4796-5p and miR-7974	SLE with or without patients with LN	Serum exosomes	Small RNA sequencing and RT-qPCR	The levels of Hsa-miR-4796-5p and hsa-miR-7974 in patients with LN are significantly increased, which can be used as better biomarkers to distinguish LN from patients with SLE, with an AUC exceeding 0.8	Chen et al. (2023a)
tsRNAs	tRF3-Ile-AAT-1 and tiRNA5-Lys-CTT-1	SLE with or without patients with LN	Urinary exosomes	Sequencing and RT-PCR	Upregulated levels of tRF3-Ile-AAT-1 and tiRNA5-Lys-CTT-1 in the urinary exosomes are measured in LN compared with SLE without patients with LN. These two tsRNAs levels are strongly associated with SLE activity	Chen et al. (2023b)

LN, lupus nephritis; HCs, healthy controls; RT-PCR, real-time polymerase chain reaction; MMP2, metalloproteinase 2; RMCs, renal mesangial cells; RTCs, renal tubular epithelial cells; SLE, systemic lupus erythematosus; RT-qPCR, real-time quantitative polymerase chain reaction; tsRNA, tRNA-derived small non-coding RNA.

Although numerous studies have confirmed that exosomes are crucial in treating kidney diseases, there are few reports concerning their application in LN patients. Evidence supporting the use of exosomes for predicting clinical responses or assessing disease activity remains limited. A long-term follow-up study was conducted with a small sample size to analyze LN patients during both the active and therapy remission stages. Notably, urinary exosomal let-7a and miR-21 were significantly downregulated in patients experiencing an active phase of LN. However, their expression levels increased following treatment-induced remission. This suggests that these biomarkers may serve as indicators for guiding the clinical stage of LN patients (Tangtanatakul et al., 2019). In another study, qPCR arrays and qRT-PCR were used to screen and identify specific miRNAs from the urinary exosomes in LN patients to predict the clinical therapeutic response. The findings revealed that the expression levels of miR-31, miR-107, and miR-135b-5p were significantly elevated in responders’ urine and kidney tissues during renal flares compared to non-responders. Among these miRNAs, miR-135b exhibited the highest predictive value. Elevated levels of these miRNAs may have contributed positively to recovery from LN (Garcia-Vives et al., 2020). An intriguing observation was reported by Somparn et al., who dynamically monitored the changes in plasma exosomal miRNA-146a levels among juvenile proliferative lupus nephritis (JPLN) patients at various stages—from onset through complete remission. Their results showed that the expression levels of exosomal miRNA-146a remained consistent at diagnosis and early treatment phases but showed a significant increase during complete remission compared to the time of diagnosis. Consequently, miRNA-146a has potential as a non-invasive biomarker for evaluating the therapeutic response in JPLN patients (Somparn et al., 2024).

### 7.6 Acute kidney injury

Acute kidney injury (AKI) is a clinical syndrome characterized by a sudden decline in renal function. AKI remains a serious global health issue, associated with high morbidity and mortality rates. Many factors can induce AKI, including trauma, major surgical procedures, sepsis, exposure to nephrotoxic agents, and ischemia/reperfusion injury. Notably, AKI is prevalent among critically ill surgical patients. The diagnosis of AKI primarily relies on the assessment of decreased urine volume and elevated serum creatinine levels. However, creatinine measurements are influenced by various confounding factors and may not effectively reflect early renal dysfunction. Furthermore, creatinine has demonstrated poor sensitivity and specificity in evaluating the severity or grade of AKI (Strauß et al., 2024). Therefore, there is an urgent need to identify new and more sensitive biomarkers for the early detection of kidney injury. Such advancements will significantly enhance our ability to monitor the progression of AKI and improve patient outcomes. Several novel indicators have been identified in blood or urine to predict and diagnose AKI (Ferreira et al., 2024). However, fewer biomarkers have been detected in the exosomes (Table 5). Exploring exosomal biomarkers for the early diagnosis of AKI is still in its infancy, and more extensive research is needed.

**TABLE 5:** Potential biomarkers in the exosomes of AKI.

Potential biomarker		Subject	Source of exosomes	Detection method	Main finding	Reference
Protein						
	Fetuin-A	AKI rats, ICU patients, and HCs	Urinary exosomes	Gel electrophoresis and mass spectrometry	Fetuin-A levels increase significantly in the early stages of different AKI models	Zhou et al. (2006)
	ATF3	AKI rats, ICU patients with AKI, CKD patients, and HCs	Urinary exosomes	Western blot	ATF3 shows a significant increase trend in the early stage of the AKI model. ATF3 has high specificity in AKI patients	Zhou et al. (2008)
	ATF3	Sepsis-AKI patients, sepsis-non-AKI patients, and HCs	Urinary exosomes	Western blot	ATF3 in urinary exosomes is an interesting sepsis-AKI biomarker with high diagnostic specificity	Panich et al. (2017)
	NHE3	AKI rats, sepsis-associated AKI patients, and HCs	Urinary exosomes	Western blot	NHE3 increases significantly in different AKI rat models and sepsis-associated AKI patients	Yu et al. (2022b)
	C3, C4, G3BP, α-2 macroglobulin, and serotransferrin	V-AKI patients and HCs	Urinary exosomes	Proteomic analysis and LC/MS	Inflammatory proteins in urinary exosomes, such as C3, C4, G3BP, alpha-2 macroglobulin, and serotransferrin, are upregulated after nephrotoxic injury in V-AKI.	Awdishu et al. (2021)
miRNAs	miR-181a-5p and miR-23b-3p	Sepsis-induced AKI model	Serum exosomes	RT-qPCR array	miR-181a-5p and miR-23b-3p are upregulated earlier than creatinine in a sepsis-induced AKI rat model	Da-Silva et al. (2022)

AKI, acute kidney injury; HCs, healthy controls; ATF3, activating transcription factor 3; CKD, chronic kidney disease; NHE3, Na^+^/H^+^ exchanger isoform 3; ICU, intensive care unit; V-AKI, vancomycin-induced AKI; G3BP, galectin-3-binding protein; LC–MS, liquid chromatography–mass spectrometry; RT-qPCR, real-time quantitative polymerase chain reaction.

Increasing evidence suggests that exosomes derived from various cell sources exhibit therapeutic effects in AKI, becoming a new research hotspot. Among these, exosomes derived from MSCs—including those sourced from bone marrow, adipose tissue, placenta, and umbilical cord—have been shown to have significant therapeutic efficacy (Table 6). In renal ischemia/reperfusion (I/R) injury models or cisplatin-induced AKI, BMSC-Exos have shown anti-apoptotic properties and the ability to reduce DNA damage in renal tubular cells through their encapsulated miRNAs (Zhu G. et al., 2019; Wang S.-Y. et al., 2023). Furthermore, it has been reported that the role of BMSCs-Exos can be genetically modified to improve the effectiveness of treatment. For example, BMSC-Exos can be engineered by overexpressing indoleamine 2, 3-dioxygenase (IDO) to produce IDO-overexpressing BMSC-Exos (BMSC-Exo-IDO) *in vitro*. The repair ability of BMSC-Exo-IDO is significantly higher than that of BMSC-Exos after renal injury (Xie et al., 2022). Adipose-derived mesenchymal stem cells (ADSCs) are pluripotent stem cells derived from adipose cells. Numerous studies have shown that ADSC-Exos play a crucial role in the treatment of AKI. ADSC-Exos can transport different nucleic acids, such as miR-342–5p (Liu W. et al., 2023) and circVMA21 (He et al., 2023), to interact with specific target molecules and exert protective effects in autophagy, apoptosis, oxidative stress, inflammation, aerobic glycolysis, and other processes. Surprisingly, one study compared the difference in efficacy between ADSC-Exos and BMSC-Exos for LPS-induced AKI. The results of the study showed that ADSC-Exos were significantly more effective than BMSC-Exos in improving kidney function and structure (Zhang et al., 2022a). According to the published literature, hUCMSC-Exos are also involved in the recovery process after kidney injury through various mechanisms. Their protective effects include the inhibition of apoptosis (Zhou Y. et al., 2013), pyroptosis (Wan et al., 2023), inflammation, the promotion of cell proliferation, autophagy (Wang et al., 2017), renal tissue repair and angiogenesis (Huang J. et al., 2022), and the maintenance of mitochondrial homeostasis (Yu et al., 2023). Human amniotic epithelial cells (hAECs) are an ideal source of stem cells. Recently, a research team from the same laboratory reported the therapeutic effects of hAEC-Exos in AKI. In models of AKI induced by different injury factors, hAEC-Exos significantly improved mortality and renal function in mice. The protective mechanisms involve several aspects, including anti-apoptosis, the regulation of angiogenesis and immune function (Ren et al., 2020), anti-inflammatory effects (Kang et al., 2021), and the maintenance of renal endothelial integrity and adhesion junctions (Chi et al., 2022). Human urine-derived stem cells (USCs) exhibit antioxidant, anti-inflammatory, and anti-apoptotic cytoprotective effects. We hypothesize that USC-Exos also exhibit a reno-protective effect on AKI. The current literature suggests that USC-Exos can exert these protective effects by transporting encapsulated nucleic acids, which act on different signaling pathway molecules. These nucleic acids include miR-216a-5p (Zhang Y. et al., 2020), miR-146a-5p (Li et al., 2020), lncRNA taurine-upregulated gene 1 (TUG1) (Sun et al., 2022), and circ DENND4C (Yang et al., 2024) In addition to stem cell-derived exosomes, which can be used as potential therapeutic tools for AKI, exosomes derived from other cell types also exhibit comparable therapeutic benefits. For example, exosomes from apical papilla stem cells (Huang T.-Y. et al., 2022), endothelial progenitor cells (Zhou et al., 2018), and human cord blood endothelial colony-forming cells (Viñas et al., 2016) have shown promising effects. These cell-derived exosomes play a role in kidney protection by alleviating oxidative stress of damaged kidney cells, reducing the expression of inflammatory factors, regulating cell apoptosis, and preventing microvascular dysfunction.

**TABLE 6:** Potential therapeutic values of different cell-derived exosomes in AKI.

Source of exosomes	Cell or animal model	Key factor	Mechanism	Reference
BMSCs	I/R rats and I/R HK-2 cells	miR-199a-3p	miR-199a-3p is delivered to renal tubule cells, downregulates Sema3A expression, and activates AKT and ERK pathways, exerting anti-apoptotic effects and alleviating kidney injury induced by I/R	Zhu et al. (2019a)
	Cisplatin-induced AKI mice and HPTC injury	Let-7b-5p	Let-7b-5p alleviates tubular epithelial cell apoptosis *via* inhibiting p53, thereby reducing DNA damage and apoptosis pathway activity	Wang et al. (2023b)
	IRI-induced AKI mice	Cell proliferation and anti-fibrotic and anti-inflammatory molecules	Accelerating the proliferation of renal tubule cells, inhibiting tubule cell apoptosis and fibrosis, promoting the secretion of anti-inflammatory factors, and thus promoting the self-repair process after kidney injury	Xie et al. (2022)
ADSCs	Sepsis-associated AKI patients and mouse models; LPS-induced HK-2 cells	miR-342–5p	ADSC-derived exosomal miR-342–5p promotes autophagy *via* the miR-342–5p/TLR9 axis, thereby protecting AKI.	Liu et al. (2023b)
	LPS-induced mice and HK-2 cells	Circ VMA21 and miR-16–5p	ADSC-derived exosomal circVMA21 inhibits LPS-induced renal tubular cell apoptosis, inflammation, and aerobic glycolysis by targeting miR-16–5p	He et al. (2023)
	LPS-induced AKI rat	Inflammatory factors and oxidative stress molecules	ADSCs-Exos inhibit inflammatory factors and oxidative stress levels, improve renal function, and protect against AKI.	Zhang et al. (2022a)
	CLP-established AKI mice model	SIRT1	ADSC-Exos activate the SIRT1 signaling pathway, inhibit kidney inflammation, improve kidney function and tissue morphology, and reduce the mortality of mice	Gao et al. (2020)
hUC-MSCs	Cisplatin-induced AKI rats and NRK-52E cells	Oxidative stress, apoptosis, and proliferation-related molecules	Ameliorating oxidative stress and apoptosis, promoting cell proliferation, and repairing cisplatin-induced renal cell damage	Zhou et al. (2013b)
	Cisplatin-induced AKI rats and NRK-52E cells	Autophagy-associated proteins	Activating autophagy and inhibiting cisplatin-induced apoptosis and inflammation, thereby reducing the renal toxicity	Wang et al. (2017)
	IRI-induced AKI rats and cisplatin-induced NRK-52E cells	Pyroptosis-associated proteins	Improving AKI by inhibiting pyroptosis	Wan et al. (2023)
	UUO-induced mice and cisplatin-induced HK-2 cells	miR-874-3p	Regulating necroptosis *via* miR-874-3p to attenuate renal tubular epithelial cell injury and enhance repair	Yu et al. (2023)
	I/R-induced AKI pigs	Apoptosis, inflammation, and angiogenesis molecules	Inhibiting I/R-induced apoptosis and necroptosis promotes renal regeneration, inhibits the expression of pro-inflammatory factors and infiltration of macrophages, and protects against the loss of renal angiogenesis	Huang et al. (2022a)
hAECs	IRI-induced AKI mice H/R-treated HK-2 cells	Anti-apoptotic, pro-angiogenetic, and immunomodulatory molecules	Reducing apoptosis, stimulating cell proliferation, preventing peritubular capillary loss, and increasing M2 macrophage polarization	Ren et al. (2020)
	CLP-established AKI mice	NF-κB and VCAM-1	Suppressing systemic inflammation and maintaining the renal endothelial integrity in septic animals	Chi et al. (2022)
	Cisplatin-induced AKI mice and HK-2 cells	TNF-α/MAPK and caspase signaling pathways	Alleviating cisplatin-induced kidney inflammation by inhibiting the TNF-α/MAPK signaling pathway and attenuating cisplatin-induced apoptosis in renal tubular epithelial cells	Kang et al. (2021)
USCs	IRI-induced AKI rats and H/R-induced HK-2 cells	miR-216a-5p	miR-216a-5p can target PTEN and regulate apoptosis *via* the AKT signaling pathway	Zhang et al. (2020b)
	IRI-induced AKI rats and H/R-induced HK-2 cells	miR-146a-5p	miR-146a-5p can target the 3′UTR of IRAK1 and subsequently inhibit the activation of NF-κB signaling and infiltration of inflammatory cells to protect renal function	Li et al. (2020)
	I/R-induced AKI mice and H/R-induced HK-2 cells	LncRNA TUG1	LncRNA TUG1 regulates the stability of ACSL4 mRNA by interacting with SRSF1 and alleviates IRI-induced AKI by suppressing ACSL4-mediated ferroptosis	Sun et al. (2022)
	I/R-induced AKI rats and H/R-induced HK-2 cells	Circ DENND4C	Promoting cell proliferation and inhibiting the activation of NLRP3 via the circ DENND4C/miR-138–5p/FOXO3a pathway, thereby reducing pyroptosis and AKI	Yang et al. (2024)
SCAPs	Cisplatin-treated NRK-52E Cells	Oxidative stress, inflammation, and cell apoptosis molecules	Protecting NRK-52E cells from cisplatin-induced AKI by inhibiting oxidative stress, inflammation, and cell apoptosis	Huang et al. (2022b)
EPCs	CLP-established AKI rats	miR-21–5p	EPCs-Exos upregulate the expression of miR-21–5p. When targeting RUNX1, miR-21–5p can improve renal function and pathological kidney tissue damage in CLP rats, inhibit inflammation and oxidative stress response, and reduce the apoptosis of kidney tissue	Zhang et al. (2021a)
	CLP-induced septic mice	miR-126–3p and 5p	EPCs-Exos prevent microvascular dysfunction and potentially improve sepsis outcomes by delivering miR-126	Zhou et al. (2018)
ECFCs	I/R-induced AKI mice	miR-486–5p	Delivery of ECFCs-Exos reduces ischemic kidney injury *via* the transfer of miR-486–5p targeting PTEN.	Viñas et al. (2016)

AKI, acute kidney injury; BMSCs, bone marrow mesenchymal stem cells; I/R, ischemia/reperfusion; Sema3A, semaphorin 3A; AKT, activated the protein kinase B; ERK, extracellular-signal-regulated kinase; HPTCs, human primary renal tubular epithelial cells; IRI; ischemia–reperfusion injury; ADSCs, adipose-derived mesenchymal stem cells; LPS, lipopolysaccharide; TLR9, Toll-like receptor 9; CLP, cecal ligation and puncture; SIRT1, sirtuin 1; hUC-MSCs, human umbilical cord mesenchymal stem cells; UUO, unilateral ureter obstruction; hAECs, human amniotic epithelial cells; H/R, hypoxia-reoxygenation; VCAM-1, vascular cell adhesion molecule-1; NF-κB, nuclear factor kappa B; MAPK, mitogen-activated protein kinase; USCs, urine-derived stem cells; PTEN, phosphatase and tensin homolog; IRAK1, interleukin-1 receptor-associated kinase 1; TUG1, taurine-upregulated gene 1; ACSL4, acyl-CoA synthetase long-chain family member 4; SRSF1, serine/arginine splicing factor 1; circ DENND4C, circDENN, domain-containing 4C; NLRP3, NLR, family pyrin domain containing 3; SCAPs, apical papilla; EPCs, endothelial progenitor cells; RUNX1, runt-related transcription factor 1; ECFCs, endothelial colony-forming cells.

### 7.7 Polycystic kidney disease

Autosomal dominant polycystic kidney disease (ADPKD) is one of the most common single-gene inherited disorders, primarily resulting from mutations in the *PKD1* gene (approximately 85%) or the *PKD2* gene (approximately 15%). The estimated prevalence of ADPKD ranges from 1/1000 to 1/400. Notably, more than half of affected individuals will progress to end-stage renal disease before the age of 60 years, requiring dialysis or kidney transplantation (Ding et al., 2021). Therefore, it is imperative to identify more sensitive early diagnostic markers and develop effective therapeutic strategies aimed at slowing the progression of ADPKD and enhancing patient prognosis.

Currently, there are limited reports on exosomal biomarkers related to ADPKD. Earlier studies utilizing proteomic analysis revealed that urinary exosomal levels of polycystin-1 (PC1) and polycystin-2 (PC2) were significantly reduced in patients with PKD1 mutations, while the levels of fibrocystic homologous transmembrane protein 2 (TMEM2) were found to be elevated. Subsequent validation through cohort studies indicated that the ratios of PC1/TMEM2 or PC2/TMEM2 could effectively differentiate individuals with PKD1 mutations from healthy controls. This suggests that these urinary exosomal proteins have practical utility in the diagnosis and monitoring of ADPKD (Hogan et al., 2015). Furthermore, the expression of the activator of G-protein signaling 3 (AGS3) is significantly increased in urinary exosomes in both animal models and patients with PKD, suggesting that AGS3 may serve as a novel biomarker for PKD (Keri et al., 2018). Another study identified up to 30 proteins that were significantly elevated in the urinary EVs of patients with ADPKD. Notably, periplakin, envoplakin, villin-1, and complements C3 and C9 are closely associated with the progression of ADPKD (Salih et al., 2016).

The diagnostic value of miRNAs derived from urinary exosomes in renal diseases has been previously demonstrated. Similarly, urinary exosomal miRNAs can be used as diagnostic tools for ADPKD. A previous study showed that miR-192-5p, miR-194-5p, miR-30a-5p, miR-30d-5p, and miR-30e-5p were significantly downregulated in the urinary exosomes of patients, murine PKD1 cystic kidneys, and human PKD1 cystic kidney tissues. This suggests their potential utility as novel biomarkers for disease progression and new therapeutic targets for ADPKD (Magayr et al., 2020). A recent study analyzed small RNAs that are differentially expressed between patients with ADPKD and healthy controls. The findings revealed that miR-320b, miR-320c, miR-146a-5p, miR-199b-3p, miR-671-5p, miR-1246, miR-8485, miR-3656, has_piR_020497, has_piR_020496, and has_piR_016271 were significantly upregulated in urine EVs from ADPKD patients. Conversely, miRNA-29c was significantly downregulated. These specific miRNAs may represent promising biomarkers for ADPKD (Ali et al., 2024). To the best of our knowledge, few studies have investigated the role of exosomes in the treatment of ADPKD. One such study conducted a proteomic analysis of urinary exosomes, which proved useful in distinguishing between rapidly progressing and slowly progressing ADPKD. Additionally, it distinguished between patients who responded favorably to tolvaptan therapy and those who did not respond adequately. The proteomic analysis of urinary exosomes provides a specific and sensitive non-invasive method for identifying disease progression rates and evaluating the efficacy of tolvaptan treatment among individuals with ADPKD—thereby indicating new avenues for personalized prognosis and management strategies related to this condition (Raby et al., 2021).

### 7.8 Renal cell carcinoma

Renal cell carcinoma (RCC) is one of the most common malignancies in the urinary system. Unfortunately, approximately 30% of patients are already in the metastatic stage at the time of diagnosis. Currently, the diagnosis of RCC primarily relies on imaging techniques and renal biopsy. However, the early clinical symptoms of RCC are non-specific and typically manifest at a later stage in disease progression, thereby missing the best opportunities for timely diagnosis and treatment. Therefore, there is an urgent need to develop non-invasive molecular detection in urine or serum to identify reliable biomarkers, which is crucial for improving the early diagnosis and treatment of RCC (Rui et al., 2023). Numerous studies have highlighted the significant roles of exosomes in several aspects of RCC, including tumorigenesis, metastasis, immune evasion, and drug response. The multiple bioactive molecules encapsulated in exosomes, such as RNA, DNA, proteins, and lipids, provide opportunities for early diagnosis and targeted interventions in RCC (Boussios et al., 2023). Table 7 shows potential exosomal biomarkers for RCC. These exosome-related molecules have also been validated.

**TABLE 7:** Potential biomarkers in the exosomes of RCC.

Potential biomarker		Subject	Source of exosomes	Detection method	Main finding	Reference
miRNAs	miR-126-3p and different combinations of miRNAs	ccRCC patients and HCs	Urinary exosomes	RT-qPCR	miR-126-3p combined with miR-449a or miR-34b-5p can significantly distinguish ccRCC patients from healthy participants	Butz et al. (2016)
	miR-30c-5p	ccRCC, prostate cancer, and bladder cancer patients, and HCs	Urinary exosomes	NGS	Sixteen urinary exosomal miRNAs are dissimilarly expressed between ccRCC patients and healthy individuals. Among them, miR-30c-5p is the only miRNA with high specificity in ccRCC.	Song et al. (2019)
	miR-204-5p	Xp11 tRCC mouse model, M-1 cells, HK-2 cells, and Xp11 tRCC cells	Urinary exosomes	qPCR array analysis and RT-qPCR	miR-204-5p levels in urinary exosomes from the Xp11 tRCC mouse model significantly increased relative to those in control mice, and these increases occurred at time points before overt tRCC development	Kurahashi et al. (2019)
	miR-210 and miR-1233	ccRCC patients and HCs	Serum exosomes	RT-qPCR	Serum exosomal miR-210 and miR-1233 are upregulated in ccRCC, independently of clinical staging	Zhang et al. (2018)
	miR-210	Renal cell lines, ccRCC patients, and HCs	Serum exosomes	RT-qPCR	Only the serum exosomal miR-210 was significantly upregulated in ccRCC patients. The area under the ROC curve was 0.8779, and the sensitivity and specificity were 82.5% and 80.0%, respectively	Wang et al. (2019)
	miR-149–3p, miR-424–3p, and miR-92a-1-5p	RCC patients and HCs	Plasma exosomes	RT-qPCR	miR-149-3p and miR-424-3p are upregulated, and miR-92a-1-5p is significantly downregulated. These three miRNAs may be novel biomarkers for the diagnosis of RCC.	Xiao et al. (2020)
	miR-224	Renal cell lines and ccRCC patients	Serum or cell culture media exosomes	RT-qPCR	Exosomal miR-224 is associated with invasion and metastasis of RCC and can be used as a biomarker reflecting the prognosis of RCC.	Fujii et al. (2017)
	miR-let-7i-5p, miR-26a-1-3p, and miR-615–3p	mRCC patients	Plasma exosomes	RT-qPCR	Multiple plasma exosomal miRNAs show an association with survival in mRCC-stage patients	Du et al. (2017)
	hsa-miR-320 d	ccRCC patients	Serum EVs	Small RNA deep sequencing and RT-qPCR	Serum EVs-derived hsa-miR-320d can be used as a prognostic biomarker for predicting ccRCC metastasis and recurrence	Xue et al. (2023)
mRNA	CUL9, KMT2D, PBRM1, PREX2, and SETD2	ccRCC, benign renal masses patients, and HCs	Serum exosomes	RT-qPCR	Establishing and validating novel exosomal mRNA-based signatures for the early detection of ccRCC and differential diagnosis of uncertain renal masses	He et al. (2022)
circular RNA	circ_400 068	RCC patients and RCC cells	Plasma exosomes	circRNA microarray and RT-qPCR	Exosomal circ_400 068 promotes the development of RCC *via* the miR-210-5p/SOCS1 axis	Xiao and Shi (2020)
snoRNAs	SNORD99, SNORD22, SNORD26, and SNORA50C	ccRCC patients and urolithiasis controls	Urine-derived EVs	RNA sequencing and qPCR	Reporting four snoRNAs as biomarkers from urine-derived EVs for the non-invasive detection of ccRCC	Grützmann et al. (2024)
Proteins	10 proteins	RCC patients and HCs	Urinary exosomes	LC–MS/MS	Identifying 261 proteins in HCs and 186 in RCC patients. Significant, reproducible differences exist in the content of some proteins between the two groups	Raimondo et al. (2013)

RCC, renal cell carcinoma; ccRCC, clear cell renal cell carcinoma; HCs, healthy controls; RT-qPCR, real-time quantitative polymerase chain reaction; NGS, next-generation sequencing; Xp11 tRCC, Xp11.2 translocation renal cell carcinoma; qPCR, quantitative PCR; mRCC, metastatic renal cell cancer; EVs, extracellular vesicles; SOCS1, suppressor of cytokine signaling 1; snoRNAs, small nucleolar RNAs.

The application of exosomes in tumor therapy has become a new research topic. Exosomes can directly transport bioactive molecules to tumor cells. This characteristic of precise release and targeted therapy makes exosomes a promising treatment for RCC. It has been demonstrated that miR-182, contained within MSC-derived exosomes, can inhibit the expression of vascular endothelial growth factor A (VEGFA) to promote the immune response of T cells, thus slowing RCC progression (Li D. et al., 2021). In 2022, Yoshino et al. found that exosomal miRNA-1 (miR-1) significantly inhibits RCC cell proliferation, migration, and invasion, suggesting that exosomal miR-1 therapy may be an effective therapeutic strategy for RCC (Yoshino et al., 2022).

### 7.9 Chronic kidney disease

Chronic kidney disease (CKD) is a global public health challenge, affecting approximately 10–15 percent of the adult population. CKD ultimately progresses to kidney failure, also referred to as ESRD, necessitating dialysis or kidney transplantation for patient survival. A critical event in the progression from CKD to ESRD is interstitial fibrosis, which is characterized by a multifactorial pathological process involving oxidative stress and an inflammatory response. Current conventional non-invasive indicators, such as blood creatinine and urea nitrogen levels, exhibit limitations in sensitivity and specificity for the early detection of renal impairment. These markers are influenced by various factors, thereby constraining their clinical application. Recent studies have highlighted that the pathophysiological changes in renal function are closely related to exosomes derived from glomeruli, tubules, interstitial cells, urine, and serum. These exosomes contain proteins and non-coding RNAs that are involved in the onset and progression of renal fibrosis. They play an important role in the early diagnosis and subsequent treatment of CKD (Yeh et al., 2024).

Increasing evidence indicates that exosomal proteins and miRNAs are expressed at abnormal levels in CKD patients and may be regarded as key biomarkers in the progression of CKD. Moreover, it is well known that CKD is characterized by an irreversible loss of renal function and is closely associated with the progression of renal fibrosis. Therefore, CKD biomarkers primarily reflect the extent of renal fibrosis. More than a decade ago, Lv et al. first investigated the value of miRNAs in urinary exosomes as potential biomarkers of renal fibrosis. Their findings revealed that the levels of urinary exosomal miR-29 and miR-200 were significantly reduced in patients with CKD. Notably, both miR-29a and miR-29c have been shown to effectively predict the degree of tubulointerstitial fibrosis, thereby distinguishing mild cases from moderate-to-severe fibrosis (Lv et al., 2013). Subsequently, results from different research teams showed that urinary exosomal miR-200b (Yu et al., 2018), miR-181a (Khurana et al., 2017), and miR-21 (Lange et al., 2019) could serve as promising biomarkers for the early detection of CKD. In addition to exosomal miRNAs, other nucleic acid components, such as non-coding RNAs, may also become a new research direction for identifying biomarkers of renal fibrosis in CKD. CircRNAs are a class of non-coding, linear RNA molecules characterized by their unique circular structure. Recently, a microarray has been used to analyze the differences in urinary exosomal circRNA expression profiles between CKD patients without renal fibrosis and those with renal fibrosis. The results showed that the expression levels of hsa_circ_0036649 correlated with the degree of renal fibrosis, showing high specificity in predicting this condition (Cao et al., 2022b). Additionally, it has also been reported that urinary exosomal hsa_circ_0008925 can act as a non-invasive biomarker for renal fibrosis. The expression level of hsa_circ_0008925 was found to be elevated in urinary exosomes from patients with renal fibrosis and exhibited a significant correlation with tubulointerstitial fibrosis and glomerular sclerosis (Cao et al., 2022a).

Exosomes also have a great therapeutic potential for CKD. Currently, the application of exosomes in CKD treatment primarily focuses on alleviating or preventing the progression of renal fibrosis. Increasing evidence suggests that MSC-derived exosomes from diverse origins have unique advantages in treating renal fibrosis. The potential therapeutic values associated with different cell-derived exosomes in patients with CKD are summarized in Table 8.

**TABLE 8:** Potential therapeutic values of exosomes derived from different cell types in CKD.

Source of exosomes	Cell or animal model	key factor	Mechanism	Reference
BMSCs	HA-VSMCs and 5/6 SNx rat models	miR-381-3p	BMSCs-Exos play anti-calcification and anti-apoptosis roles in CKD by delivering enclosed miR-381–3p, directly targeting NFAT5 mRNA	Liu et al. (2022c)
	UUO mice model and TCMK-1 cells	miR-21a-5p	BMSCs-Exos significantly ameliorate UUO-induced renal fibrosis by inhibiting glycolysis in TECs	Xu et al. (2022)
	UUO mice model and pericytes	miR-34c-5p	BMSCs-Exos deliver miR-34c-5p into pericytes, fibroblasts, and macrophages, and miR-34c-5p downregulates CF to inhibit multiple signaling pathways, thereby ameliorating multiple cellular activations and RIF	Hu et al. (2022)
	UUO mice model and HK-2 cells	miR-374a-5p	Exosomal miR-374a-5p inhibited the progression of renal fibrosis by regulating the MAPK6/MK5/YAP axis	Liang et al. (2022a)
	UUO mice model and NRK-52E cells	Let-7i-5p antagomir	Exosomal anti-let-7i-5p from BMSCs exerts anti-fibrotic effects in TGF-β1-induced fibrogenic responses in NRK52E cells *in vitro* and in the UUO-induced renal fibrosis model *in vivo*	Jin et al. (2021)
	UUO mice model and NRK52E cells	miR-let7c	The anti-fibrotic function of engineered MSCs can selectively transfer miR-let7c to damaged kidney cells and significantly downregulate the expression of fibrosis genes	Wang et al. (2016)
	5/6 SNx rat models	klotho activity	BMSCs-Exos may protect against kidney injury, probably by regulating klotho activity and expression	Wan et al. (2022)
	5/6 SNx rat models and HRPTEpics	Smurf 2/Smad 7 axis	BMSCs-Exos partially inhibit renal fibrosis both *in vivo* and *in vitro* by regulating the Smurf 2/Smad 7 axis	Liu et al. (2022b)
	5/6 SNx mouse models	SIRT6 and HMGB1	BMSCs-Exos inhibit high phosphate-induced aortic calcification and ameliorate renal function *via* the SIRT6-HMGB1 deacetylation pathway	Wei et al. (2021)
hUC-MSCs	UUO rat model and NRK52E cells	YAP activity	hUC-MSCs-Exos can increase CK1δ and β-TRCP expression and promote YAP ubiquitination and degradation, which inhibits ECM deposition and alleviates renal fibrosis	Ji et al. (2020)
	UUO mice model and RAW264.7 cells	ARNTL	hUC-MSCs-Exos reduce renal fibrosis in UUO mice by inhibiting MMT and may be associated with regulating ARNTL expression	Guo et al. (2024)
	HK-2 cells	miR-335–5p	hUC-MSCs-derived exosomal miR-335–5p attenuates the inflammation and EMT of HK-2 cells by reducing ADAM19 protein levels	Qiu et al. (2022)
ADSCs	CKD mouse model	Molecules associated with inflammation and fibrosis	ADSCs-Exos inhibit renal fibrosis by regulating apoptosis and fibrosis-related cell proliferation, reducing the severity of CKD, and restoring renal function by increasing the levels of aquaporins 2 and 5	Yea et al. (2021)

CKD, chronic kidney disease; BMSCs, bone marrow mesenchymal stem cells; SNx, subtotal nephrectomy; HA-VSMCs, human aortic smooth muscle cells; NFAT5, nuclear factor of activated T cells 5; UUO, unilateral ureter obstruction; TECs, tubular epithelial cells; CF, core fucosylation; RIF, renal interstitial fibrosis; TGF-β1, transforming growth factor beta 1; HRPTEpCs, human renal proximal tubular epithelial cells; SIRT6, sirtuin 6; HMGB1, high mobility group box 1; hUC-MSCs, human umbilical cord mesenchymal stem cells; CK1δ, casein kinase 1δ; YAP, yes-associated protein; ECM, extracellular matrix; MMT, macrophage-to-myofibroblast transformation; EMT, endothelium-mesenchymal transition; ADAM19, metalloproteinase domain-containing protein 19; ADSCs, adipose-derived mesenchymal stem cells.

### 7.10 Kidney transplantation

Kidney transplantation is an effective treatment for end-stage renal disease, significantly improving patients’ quality of life and prolonging survival. Although kidney transplantation is an effective treatment, recipients still often encounter challenges such as rejection, adverse effects from immunosuppressive medications, and delayed recovery of the transplanted kidney’s function. Although renal biopsy is considered the gold standard for the diagnosis of transplant rejection, it is an invasive procedure with a high cost. Consequently, there is a pressing need for non-invasive biomarkers with high sensitivity and specificity to monitor, diagnose, and detect allograft rejection at an early stage (Ramalhete et al., 2024). Exosomes have been identified to play an important role in allograft rejection (Saravanan et al., 2023). Currently, various exosomal biomarkers are used to assess response after kidney transplantation, as detailed in Table 9.

**TABLE 9:** Potential biomarkers in the exosomes of kidney transplantation.

Potential biomarker		Subject	Source of exosomes	Detection method	Main finding	Reference
miRNAs	Hsa-miR-21-5p, hsa-miR-31-5p, and hsa-miR-4532	AR, STA, and OGI recipients	Urinary exosomes	RT-qPCR	Identifying 29 urinary exosomal microRNAs as the candidate biomarkers of AR. The combination of hsa-miR-21-5p, hsa-miR-31-5p, and hsa-miR-4532 has higher performance for AR identification	Seo et al. (2023)
	miR-21, miR-210, and miR-4639	Kidney transplant recipients and HCs	Plasma exosomes	RT-qPCR	miR-21, miR-210, and miR-4639 in plasma exosomes correlate closely with eGFR. The combined diagnostic value of the three miRNAs is better than that of single or double indicators	Chen et al. (2020)
	miR-21	Post-kidney transplantation patients	Plasma exosomes	RT-qPCR	Plasma exosome miR-21 is an interesting non-invasive IF/TA grade II/III biomarker that is better than the current clinical biomarkers of renal function	Saejong et al. (2022)
mRNA	Exosomal mRNA signature	Kidney transplant recipients	Urinary exosomes	RT-PCR	Providing the high-performance characteristics of urinary exosome RNA to discriminate active rejection from no rejection and ABMR from TCMR	El Fekih et al. (2021)
	Gp130, SH2D1B, TNFα, and CCL4	Kidney transplant recipients	Plasma exosomes	RT-qPCR	In the plasma exosomes of AMR patients, mRNA levels of several genes are significantly increased. The gene combination of gp130, SH2D1B, TNF-α, and CCL4 is the best	Zhang et al. (2017)
Proteins	349 exosomal proteins	Kidney transplant patients with and without AR	Urinary exosomes	LC–MS/MS	Three hundred forty-nine proteins are identified in urine exosomes. Among them, 11 proteins are more abundant in urine samples from patients with AR, and three are exclusive to the urine exosome fraction	Sigdel et al. (2014)
	Tetraspanin-1 and hemopexin	Kidney transplant recipients including STA and TCMR	Urinary exosomes	Nano-UPLC–MS/MS	Seventeen proteins were increased in TCMR patients. Among the five TCMR candidate biomarkers, only tetraspanin-1 and hemopexin perform better	Lim et al. (2018)
	SYT17	CKD patients, kidney transplant patients, and HCs	Urinary exosomes	Western blotting	Urinary exosomal SYT17 levels are significantly elevated in the CAAMR group compared to other histology groups	Takada et al. (2020)
	AZGP1	Kidney transplant recipients including CAMR and LGS	Urinary exosomes	LC–MS analysis and Western blotting	AZGP1, in particular, was found to be a CAMR-specific proteomic biomarker	Jung et al. (2020)
	CST3 and LBP	Kidney transplant recipients including ABMR, TCMR, BKVN, and NOMOA	Urinary exosomes	Nano-LC-ESI-MS/MS analysis	Urinary exosomal CST3 and LBP levels are significantly higher in the ABMR group	Kim et al. (2022)

AR, acute rejection; STA, stable graft function; OGIs, other graft injuries; RT-qPCR, real-time quantitative polymerase chain reaction; HCs, healthy controls; IF/TA, interstitial fibrosis and tubular atrophy; RT-PCR, real-time polymerase chain reaction; TCMR, T-cell-mediated rejection; ABMR, antibody-mediated rejection; AMR, antibody-mediated rejection; nano-UPLC–MS/MS, nano-ultra performance liquid chromatography–tandem mass spectrometry; SYT17, synaptotagmin-17; CKD, chronic kidney disease; CAAMR, chronic active antibody-mediated rejection; CAMR, chronic active antibody-mediated rejection; AZGP1, zinc-alpha-2-glycoprotein; LGS, long-term graft survival; CST3, cystatin C; LBP, lipopolysaccharide-binding protein; BKVN, BK virus nephropathy; NOMOA, no major abnormality.

Immunosuppressive agents are most commonly used to prevent immune rejection in renal transplantation; however, they cause immune-mediated damage and adverse effects. Therefore, studies on using exosomes for preventing and mitigating immune rejection in renal transplantation have become an area of significant interest. Recent investigations utilizing a mouse kidney transplantation model have focused on the antagonistic non-protein coding RNA (DANCR), a long-chain non-coding RNA (lncRNA) found in bone marrow mesenchymal stem cell-derived exosomes. This lncRNA has been shown to promote immune tolerance in kidney transplantation by downregulating sirtuin-1 (SIRT1) expression and promoting the differentiation of CD4^+^ T-cells into Treg-cells (Wu X. et al., 2022). Renal ischemia–reperfusion injury (IRI) is a common clinical symptom of renal transplantation. Exosomes derived from umbilical cord mesenchymal stem cells (UC-MSC-Exos) exhibit certain advantages in treating IRI in transplanted kidneys. A recent study conducted by Wu et al. explored how UC-MSC-Exos ameliorate renal transplant-related IRI, identifying miR-19b as a critical factor. miR-19b was found to reduce the expression of the metabolic enzyme PDXK, increase levels of the metabolite pyridoxine, and ultimately reduce apoptosis in I/R-HK2 cells by targeting GSK3β, thereby providing a new therapeutic target for IRI after kidney transplantation (Wu et al., 2024). In summary, there is limited research regarding the role of exosomes in treating and recovering renal function post-kidney transplantation. Further comprehensive studies are warranted to elucidate the therapeutic potential of exosomes more thoroughly.

## 8 Limitation of exosomes

Although exosome research has made remarkable progress, several challenges still exist. These issues include the following: first, although there are many methods for the production, isolation, and purification of exosomes, most of these procedures are complex and time-consuming, making it difficult to meet the needs of rapid clinical detection. Therefore, more convenient methods must be developed and improved. Moreover, various studies used different methods, complicating the reproducibility of results. Therefore, it is essential to establish more stringent protocols for the isolation and purification of exosomes to achieve unified standardization that allows for comparability and reproducibility across different studies. Second, the study of exosome biomarkers in kidney diseases is mostly a phenomenon description, which is still in the initial stage. The biomarkers used for diagnosing kidney disease primarily stem from exosome-derived miRNAs and proteins. However, many studies have relied on smaller validation sample sizes. Thus, further validation using larger clinical samples is necessary to ascertain the disease specificity of these biomarkers. Additionally, the complexity inherent in various types of kidney diseases must be taken into account. Different types of kidney diseases may exhibit similar trends in biomarker changes, which can increase the difficulty of using exosomes as biomarkers of kidney diseases. Moreover, the coexistence of multiple diseases may further challenge the clinical application of exosomes. Third, current research studies on the role of exosomes in treating kidney diseases have predominantly focused on MSC-derived exosomes. However, the experimental protocols for extracting exosomes from mesenchymal stem cells still lack standardization, and there are no effective methods for the large-scale production and purification of exosomes. Fourth, there are still some other issues to be considered in the clinical treatment of kidney diseases with exosomes. More preclinical studies and pharmacological dynamic monitoring are needed to reveal the optimal dose, mode of administration, time point, and duration for exosome therapy. More importantly, many current *in vitro* studies, animal experiments, and preclinical studies do not provide a comprehensive analysis of the long-term efficacy and potential side effects of exosomes in the treatment of kidney disease. For instance, while MSC-derived exosomes are known for their immunomodulatory and anti-fibrotic effects, their immunogenicity and pro-tumorigenic risks in high-dose or long-term applications remain unclear. This is especially relevant for chronic kidney disease patients who may require repeated dosing, where issues such as immune tolerance or side effect accumulation could arise. In addition, the immune response evaluations in preclinical models of ADSC-derived exosomes and the exploration of strategies for targeted delivery to reduce systemic side effects require more attention and research. Fifth, the therapeutic effects of exosomes have primarily been demonstrated through experiments utilizing animal and cell models, resulting in a notable absence of clinical research evidence. Nevertheless, it is encouraging to note that research into the roles of exosomes in disease treatment is progressing rapidly. We believe that the application of exosome-based therapies will gradually transition from basic research to clinical practice.

## 9 Future directions

### 9.1 Application of engineered exosomes

Engineered exosomes represent a class of modified extracellular vesicles derived from natural exosomes through bioengineering processes. This approach overcomes some of the inherent limitations of natural exosomes, such as unpredictable targeting, short half-lives, and variability in the composition of the functional cargoes. Engineered exosomes exhibit enhanced drug delivery efficiency and specificity, enabling more effective delivery to target cells or tissues. The preparation techniques for engineered exosomes primarily encompass surface modification and content modification. Surface modification involves altering the exosome surface via genetic engineering or chemical methods to improve targeting and stability. For instance, targeting peptides can be conjugated to exosomes to facilitate specific binding to target cells. Content modification integrates therapeutic agents, such as small molecule drugs or nucleic acids, into exosomes for targeted drug delivery. This method increases local therapeutic agent concentration while minimizing side effects. Common methods for content modification include co-incubation, due to its simplicity, and physical methods like ultrasound, electroporation, freeze–thaw cycles, and extrusion, although these may compromise exosomal membrane integrity to varying degrees (Fan et al., 2024). With ongoing technological advancements, the integration of gene editing technologies with exosomes is emerging as a promising direction for disease treatment. The synergistic effect of CRISPR/Cas9 gene editing and exosome therapy offers the potential for highly targeted and personalized therapeutic strategies (Dara et al., 2024). Currently, engineered exosomes show significant promise across various diseases (Tian et al., 2023). However, their application in kidney disease treatment remains nascent and limited. It is reported that Wang L. et al. (2024) demonstrated the renal protective properties of exendin-4-enriched exosomes derived from human umbilical cord mesenchymal stem cells in diabetic nephropathy. Additionally, techniques such as CRISPR/Cas9-mediated gene editing or electroporation-based siRNA loading (e.g., targeting TGF-β1) hold considerable potential for anti-fibrotic treatments in chronic kidney disease.

### 9.2 Enhancing the transformation of basic research into clinical practice

In recent years, an increasing number of models have been used to investigate the protective role of exosomes in renal diseases. However, to date, the majority of studies have been confined to cellular and animal models. There are several factors limiting clinical trials of exosomes, one of which is the low separation efficiency and purity of exosomes. Additionally, ethical concerns associated with exosome research pose another potential barrier to their clinical translation. There is now an urgent need for clinical trials to assess the efficacy and safety of exosome therapy. In particular, large-scale clinical trials and long-term follow-up studies are essential for elucidating the therapeutic potential of mesenchymal stem cell-derived exosomes in nephropathy treatment. In addition, future studies should pay more attention to the clinical translation of engineered exosomes in the treatment of kidney diseases. Determining the optimal dose, timing, route of administration, and effective measures to avoid side effects of engineered exosome therapy are important issues faced by researchers and essential steps in advancing exosomes to clinical practice.

### 9.3 Application of combination therapies

Although MSC-derived exosomes have shown significant therapeutic effects in various kidney diseases, these treatments are often single, and their efficacy still needs to be improved. Future research studies should investigate the potential of combining exosome therapy with other therapeutic approaches. For instance, integrating exosome therapy with pharmacological agents or bioactive molecules or utilizing exosome engineering to deliver multifunctional exosomes carrying anti-inflammatory factors (e.g., miR-26a-5p) and anti-fibrotic molecules (e.g., siRNA) can simultaneously achieve dual therapeutic effects against inflammation and fibrosis in diabetic nephropathy and chronic kidney disease.

### 9.4 Precise classification and extraction of kidney specific exosomes

Exosomes have different cell origins and can be obtained from a variety of cells. The surface proteins and functions of exosomes derived from different cells are different. If the classification of exosomes is not accurate, their clinical application and development can be limited to a certain extent. In kidney disease, urinary exosomes used for diagnosis originate from diverse sources such as glomerular endothelial cells, mesangial cells, podocytes, and renal tubular epithelial cells. The isolation and identification of disease-specific exosomes using specific molecular markers to improve the specificity of disease diagnosis remains an urgent problem.

## 10 Summary

In conclusion, research on the application of exosomes in renal diseases is currently experiencing rapid advancement. Their potential for diagnosis, treatment, and prognosis monitoring holds great promise. With ongoing technological progress and comprehensive investigations, exosomes are expected to emerge as a crucial tool in the management of renal diseases, thereby significantly enhancing patients’ health outcomes and quality of life.
